# Developing medical devices with emerging technologies: trends, challenges, and future directions

**DOI:** 10.12688/f1000research.154869.1

**Published:** 2024-09-04

**Authors:** Achraf akkaoui, Yassine ZAHIDI, Mohamed El Moufid, Wafaa DACHRY, Hassan GZIRI, Hicham Medromi

**Affiliations:** 1 Engineering, Industrial Management and Innovation (EIMI), Hassan 1st University Faculty of Science and Technology Settat, Settat, Chaouia-Ouardigha, Morocco; 2 Foundation for Research Development and Innovation in Science and Engineering, Casablanca, Grand Casablanca, Morocco; 3 The International Academy of Scientific Francophone (IASF), Rabat, Morocco

**Keywords:** Artificial Intelligence, Internet of Medical Things, Augmented Reality, Cybersecurity, Medical Devices, Health care, Embedded systems

## Abstract

This paper examines the rapid advancements and integration of emerging technologies in the medical field, particularly focusing on Artificial Intelligence (AI), the Internet of Medical Things (IoMT), Augmented Reality (AR), and cybersecurity. The study leverages data from Scopus and Web of Science databases to highlight the trends, challenges, and future directions in the development of medical devices. Significant progress has been made in enhancing patient care through the integration of AI and IoMT, which facilitate predictive analytics, personalized treatment plans, and real-time data monitoring. AR is transforming medical training and surgical precision, while cybersecurity measures are becoming increasingly vital to protect sensitive health data. Despite these advancements, the field faces challenges such as data privacy concerns, infrastructure limitations, and interoperability issues. The study also explores Africa’s contributions, with a particular emphasis on Morocco’s emerging role in this technological landscape. Three major research clusters identified include AI and AR, IoT and cybersecurity, and embedded systems, each playing an important role in the evolution of medical technologies. By analyzing publications from 2010 to 2024, the paper provides insights into the current state and future potential of advanced medical technologies, aiming to offer a foundation for further research and innovation in this rapidly evolving field.

## 1. Introduction

The rapid advancement of technology has significantly impacted the medical field, introducing innovative solutions that enhance patient care. Among these technologies, Artificial Intelligence (AI) and the Internet of Medical Things (IoMT) stand out due to their transformative potential. AI, with its capabilities in machine learning, deep learning, natural language processing, and data analysis, is revolutionizing how medical data is interpreted and utilized. It enables predictive analytics, and personalized treatment plans, which contribute to more accurate diagnoses and improved patient outcomes. ^1^^–^^4^ Meanwhile, IoMT connects medical devices through the internet, enabling real-time data collection, monitoring, and analysis, thus improving the efficiency and accuracy of healthcare delivery. ^5^^–^^9^

The integration of these technologies in medical devices comes with challenges such as data privacy and security. These challenges are paramount concerns, given the sensitive nature of health information. Cybersecurity measures must be robust to protect against breaches and unauthorized access. ^10^^–^^12^ Infrastructure limitations, such as the need for reliable internet connectivity and advanced computational resources, also pose significant barriers. Furthermore, interoperability issues between various devices and systems complicate the seamless integration of these technologies into existing healthcare frameworks. ^13^^,^^14^ Despite these barriers, the potential benefits of AI, IoMT, augmented reality (AR), big data analytics, and cybersecurity measures are driving substantial research and development efforts.

This study explores the evolution and current landscape of advanced technologies in the medical field by conducting an academic publications analysis from 2010 to 2024. Utilizing data from two prominent databases, Scopus, and Web of Science, we seek to identify key trends, highlight significant contributions, and map the global distribution of research in this domain. The selection of these databases was based on their extensive coverage of peer-reviewed literature and their relevance to science, technology, and medicine.

Our methodology involves systematic keyword-based searches, merging datasets, and eliminating duplicates using the Bibliometrix library in R, followed by data analysis and visualization with Biblioshiny and VOSviewer. ^15^ This approach ensures a thorough and insightful examination of the data, highlighting key trends and developments in the field. By understanding these dynamics, we can gain a clearer understanding of the progress made and the challenges ahead in integrating advanced technologies into medical devices. Through this study, we strive to deliver valuable insights into the trends and future directions of medical device technologies, offering a foundation for further research and innovation in this field.

This article seeks to afford answers to questions regarding the integration of advanced technologies in medical devices:

RQ1: What are the contributions and advancements made by different regions, particularly Africa, in this technological landscape?

RQ2: What are the current trends and advancements in the integration of advanced technologies in medical devices?

RQ3: What are the major challenges and barriers to the implementation of these advanced technologies in the medical field?

In the first section, we will outline the methodology and materials used for our study, detailing the data collection process and the analytical tools employed. The second section will give an overview of the advanced technologies covered in this research, including AI, IoMT, AR, Big Data Analytics, and Cybersecurity. The third section will delve into the analysis of the collected data, presenting key findings and trends. Finally, we will discuss the implications of these advancements and the challenges ahead in integrating these technologies into medical devices, concluding with insights into future research directions and potential developments in this rapidly evolving field.

## 2. Methods

In this article, we reviewed academic publications to examine the progress and state of advanced medical technologies, specifically AI and IoMT. Our analysis included data from Scopus and Web of Science, which were selected because they have a significant amount of peer-reviewed literature in science, technology, and medicine.

We initiated our data collection with a search using the query: ((“medical devices” AND (“artificial intelligence” OR “internet of medical things” OR “deep learning” OR “Internet of Things” OR “machine learning” OR “Augmented reality”)). we refined our dataset by applying inclusion criteria based on specific keywords such as “Artificial Intelligence” or “Internet Of Things” or “Medical Devices” or “Health Care” or “Machine Learning” or “Biomedical Equipment” or “Big Data” or “Medical Device” or “Deep learning” or “Wearable Devices” or “Internet Of Medical Things” or “Cloud Computing” or “Augmented Reality” or “Wearable Medical Devices” or “Virtual Reality” or “Real-time”. This refinement was geared towards focusing on the specific technologies and their applications in medical devices.

The search was limited to articles published between 2010 and 2024 to capture the most recent and relevant developments in the field. This timeframe was selected due to the significant technological advancements since 2010 have transformed the landscape of medical devices. To eliminate redundancy and ensure the uniqueness of the records, we used the Bibliometrix ^16^ library in R, which facilitated the merging of datasets from Scopus and Web of Science and the removal of duplicate records.

**Figure 1.**
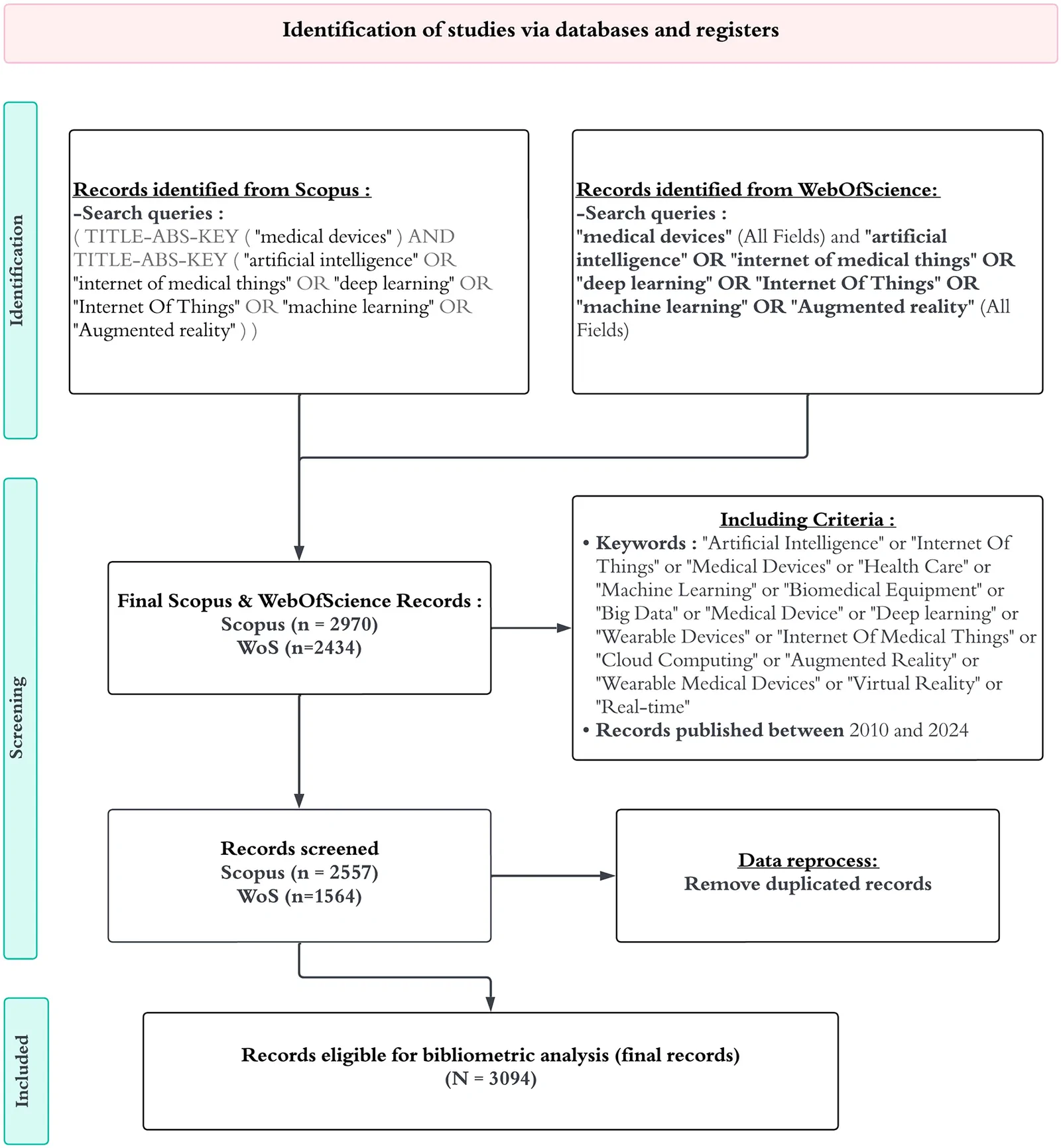
PRISMA diagram for data collection and refinements.

For the bibliometric analysis, we employed the PRISMA method ^17^ to ensure a systematic and transparent approach to data collection. The method is presented in Figure 1. Additionally, we used Biblioshiny from Bibliometrix ^16^ for advanced data analysis and visualization, as well as VOSviewer ^15^ for constructing and visualizing bibliometric maps using the VOS (Visualization of Similarities) mapping technique, which uses mathematical algorithms to visualize relationships between items. These tools collectively enabled a thorough and insightful examination of the data, highlighting key trends and developments in the field.

Table 1 gives an overview of the bibliometric data for the study, covering 2010 to 2024. The scope encompasses 3,094 documents from 1,747 sources, with a remarkable annual growth rate of 27.8%. Each document has an average of 13.92 citations, and it is rated as mean by the average lifespan of 3.24 years. The dataset holds contributions from 10,695 authors, with a substantial portion of them being involved in international collaboration (16.29%). The documents have a wide range of types, with most of them being articles (1,405), followed by conference papers (580), reviews (376), and book chapters (165). Through the analysis of this extensive data, we can establish a solid base for exploring trends and progress in medical device technologies.

**Table 1.** Main information of final records.

Description	Results
MAIN INFORMATION ABOUT DATA	
Timespan	2010:2024
Sources (Journals, Books, etc.)	1747
Documents	3094
Annual Growth Rate %	27,8
Document Average Age	3,24
Average citations per doc	13,92
References	115522
DOCUMENT CONTENTS	
Keywords Plus (ID)	11335
Author's Keywords (DE)	7636
AUTHORS	
Authors	10695
Authors of single-authored docs	216
AUTHORS COLLABORATION	
Single-authored docs	231
Co-Authors per Doc	4,74
International co-authorships %	16,29
DOCUMENT TYPES	
Article	1405
Book chapter	165
Conference paper	580
Review	376

## 3. Advanced technologies overview

### 3.1 Artificial intelligence

Artificial Intelligence (AI) simulates human intelligence processes through programmed machines capable of thinking and learning. It includes diverse subfields such as machine learning, where algorithms autonomously improve from data without explicit programming; deep learning, which processes data through complex neural networks; natural language processing (NLP), enabling machines to understand and replicate human language; and computer vision, which interprets and acts on visual data from the world around us. In the healthcare sector, AI applications range from robotic surgeries offering precision beyond human capabilities to virtual nursing assistants reducing unnecessary hospital visits and burden on medical staff. ^18^^–^^20^ In finance, AI is crucial for real-time transaction monitoring and fraud detection, enhancing security and customer experience. AI’s integration into autonomous vehicles is transforming transportation, improving safety, and optimizing traffic management, illustrating its transformative impact across various industries. ^1^^,^^2^

### 3.2 Internet of things and internet of medical things

The Internet of Things (IoT) integrates physical objects embedded with sensors, software, and other technologies that connect and exchange data over the internet. This connectivity enables real-time monitoring, control, and optimization of processes across numerous sectors. In manufacturing, IoT technologies facilitate predictive maintenance, reducing downtime by predicting equipment failures before they occur. ^5^^,^^6^^,^^12^

In the healthcare realm, the Internet of Medical Things (IoMT) leverages IoT technologies to enhance patient monitoring and care. Devices such as smart inhalers and wearable fitness trackers collect health data, offering insights into patient habits and improving chronic disease management. Further, IoMT supports remote patient monitoring, allowing doctors to make informed decisions remotely and efficiently. The expansion of IoMT is set to revolutionize healthcare delivery, making it more patient-centered and data-driven. ^7^^–^^9^^,^^21^^–^^23^

### 3.3 Augmented reality and virtual reality

Augmented Reality (AR) and Virtual Reality (VR) are reshaping how we interact with digital information and the world around us. AR enhances the real world with digital overlays, providing valuable information in real-time. This technology has profound applications in medical training, where AR goggles display patient data during procedures, allowing for more precise and safer surgeries. In education, AR apps bring complex academic concepts to life, enhancing understanding and retention. VR creates immersive virtual environments, extensively used in therapy and rehabilitation. It offers controlled and intensive therapy sessions for patients recovering from strokes or injuries, significantly speeding up the recovery process without the physical constraints of the real world. In entertainment, VR games and experiences allow users to immerse themselves completely in digital worlds, providing new forms of engagement. ^14^^,^^24^^–^^28^

### 3.4 Big data analysis

Big Data Analysis examines vast sets of data to uncover hidden patterns, trends, and insights. This analysis is essential in sectors like healthcare, where big data is used to analyze patient information to predict disease patterns and treatment outcomes, leading to more effective interventions tailored to individual needs. In urban planning, big data helps manage everything from traffic flows to energy use, making cities smarter and more efficient. Retailers use big data to analyze consumer behavior, customizing marketing strategies to individual preferences and boosting sales. The growing capabilities in big data analytics continue to push the boundaries, making significant impacts on societal efficiency and economic activities. ^3^^,^^4^

### 3.5 Cybersecurity

As digital technologies permeate all aspects of life, cybersecurity has become a critical pillar of the digital economy, essential for protecting sensitive data and preventing cyber threats. In healthcare, robust cybersecurity measures protect patient records and ensure the integrity of medical devices. In the financial sector, cybersecurity defends against sophisticated cyber-attacks aiming to breach data and disrupt services. The increasing adoption of IoT devices has also expanded the scope of cybersecurity, necessitating the development of advanced strategies to secure interconnected devices and networks. Emerging trends in cybersecurity include the use of artificial intelligence to detect unusual patterns and behaviors quickly and accurately, offering proactive threat prevention in an increasingly interconnected world ^10^^–^^12^

## 4. Analysis of collected data

The integration of advanced technologies in medical devices and healthcare systems has significantly transformed patient care, diagnostics, and treatment methods. This transformation is driven by the increasing adoption of innovations such as AI, machine learning, and the IoT, which offer immense potential to improve healthcare outcomes. The following sections delve into specific aspects of this technological integration, examining the trends, global contributions, and research focuses that are shaping the future of healthcare.

### 4.1 Words’s frequency over time

**Figure 2.**
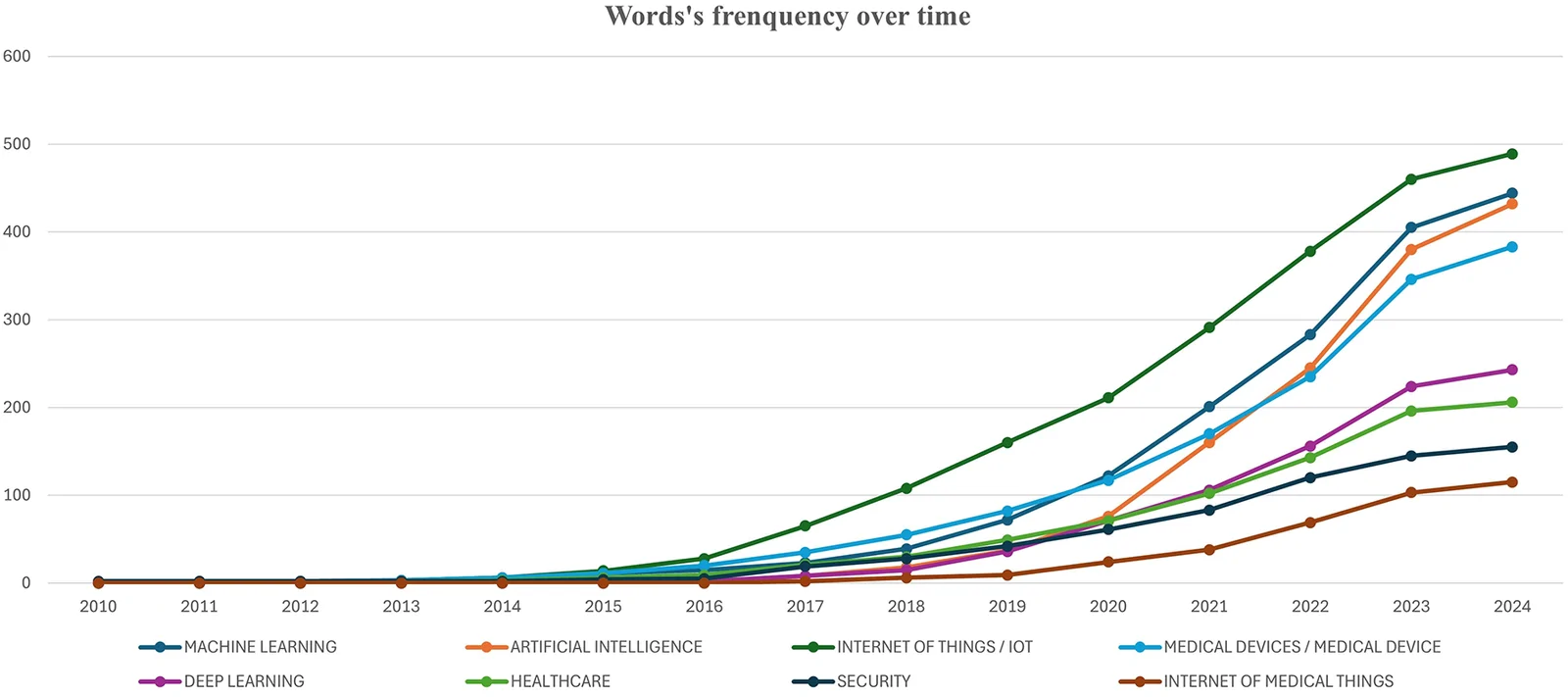
The frequency of various keywords overtime.

Figure 2, which was created using Biblioshiny from the Bibliometrix for data extraction and analysis, with further visualization adjustments made in Microsoft Excel, illustrates the frequency of various keywords related to advanced technologies in healthcare over the period from 2010 to 2024. Notably, terms such as “Machine Learning,” “Artificial Intelligence,” “Deep Learning,” “Healthcare,” “Security,” “Internet of Things (IoT),” “Medical Devices,” and “Internet of Medical Things” have shown a marked increase in usage, particularly from 2016 onwards. “Healthcare” and “Internet of Things/IoT” demonstrate a steep upward trend, indicating a growing interest and integration of these technologies in the medical field. Similarly, “Machine Learning” and “Artificial Intelligence” have seen substantial growth, reflecting their expanding role in healthcare innovation. The consistent rise in “Security” highlights the increasing focus on safeguarding sensitive medical data in the era of interconnected devices. Overall, the graph underscores the rapid adoption and emphasis on these advanced technologies, emphasizing their significance in modernizing healthcare and medical device development.

### 4.2 Countries’ scientific production

**Figure 3.**
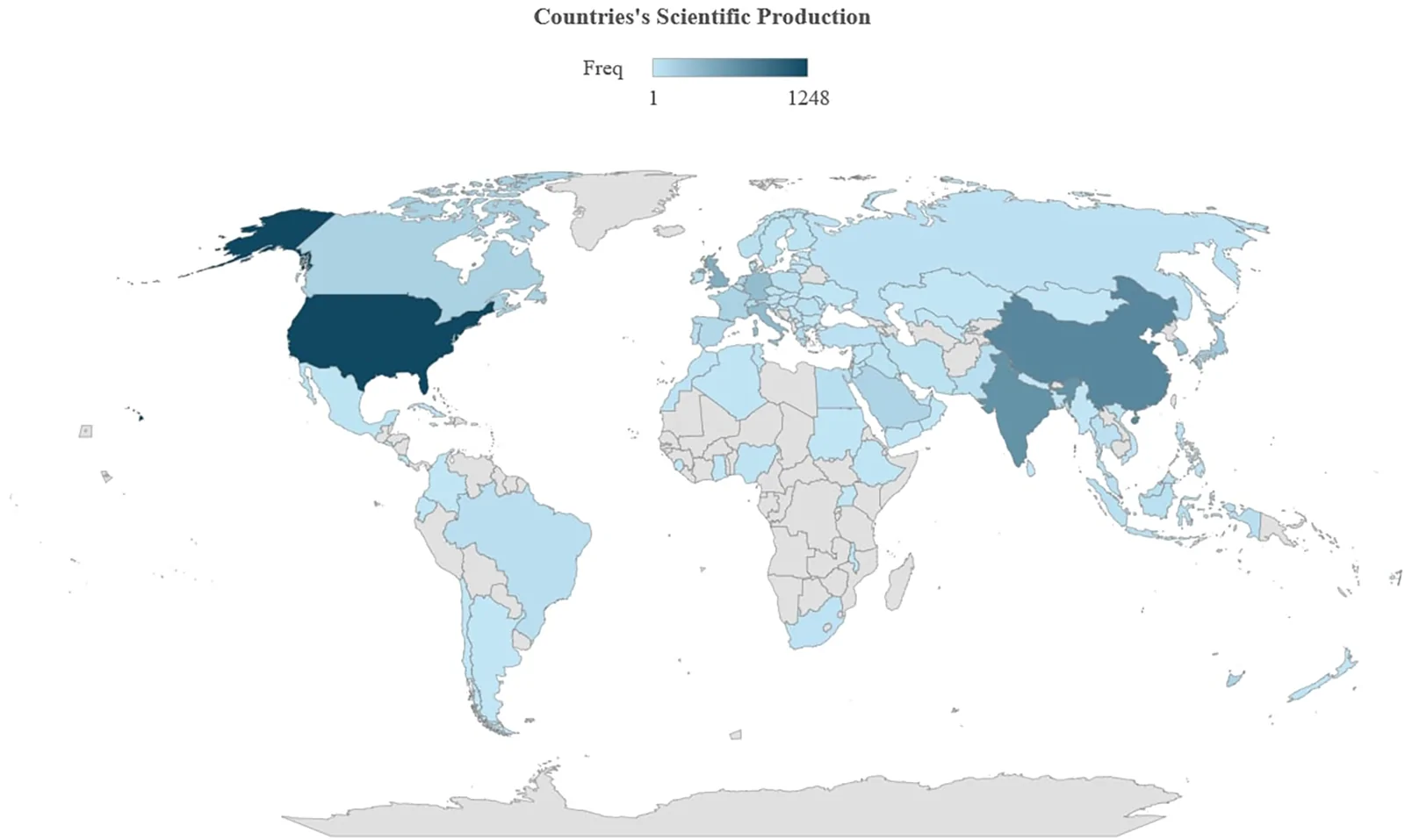
Countries' scientific production of advanced technologies for medical devices.

Figure 3, which was made by utilizing Biblioshiny for initial data handling and Microsoft Excel for the final graphical presentation, illustrates the global distribution of scientific production related to advanced technologies in healthcare. The United States leads with the highest frequency of scientific output (1248 publications), followed by China (737), India (654), and the United Kingdom (437). Other significant contributors include Italy, Germany, South Korea, and Japan. The map visually stands for these data, with darker shades indicating higher levels of scientific production. Africa’s scientific production, while less prolific compared to other regions, shows emerging contributions from countries such as Egypt (54 publications), South Africa (22), and Morocco (22). Morocco is noteworthy for its role in advancing healthcare technologies within the African continent, proving its commitment to scientific research and development in this field. This distribution highlights the concentration of research activities in North America, Asia, and parts of Europe, reflecting the regions’ strong focus on advancing healthcare technologies. The data underscores the pivotal role of these countries in driving innovation and contributing to the global body of knowledge in this field, while also indicating the growing participation of African nations in this critical area of research.

### 4.3 Keyword clusters analysis

**Figure 4.**
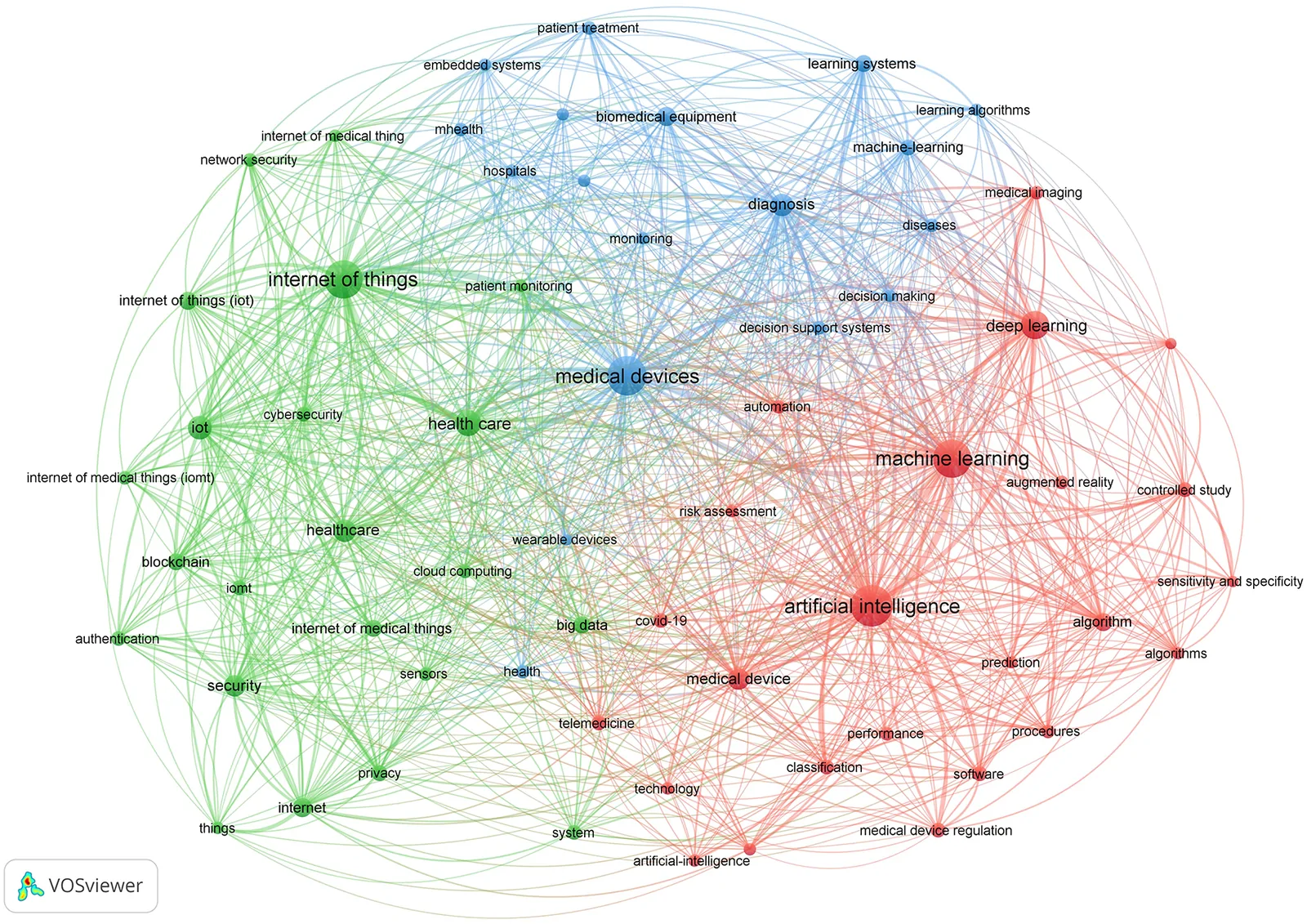
Visualization of key research clusters in advanced medical technologies using VOSviewer.

VOSviewer is a powerful tool that allows for the visualization and examination of the structure and progression of scientific research. When used to analyze articles related to advanced medical technologies, VOSviewer ^15^ can identify and map significant research clusters. As shown in Figure 4, these clusters highlight three focus areas.

Figure 4 is a visualization of Key Research Clusters in Advanced Medical Technologies, analyzed using VOSviewer ^15^ with a minimum co-occurrence of keywords set at 50, resulting in 66 final keywords. This method provides a comprehensive overview of current research and emerging trends, offering valuable insights for both researchers and practitioners. The artificial intelligence (AI) and augmented reality (AR) cluster, the red cluster, encompasses research on the integration of AI and AR technologies to enhance the functionality and effectiveness of medical devices, improving diagnostic accuracy and patient outcomes. The Internet of Things (IoT) and Cybersecurity cluster, the green cluster, focuses on the intersection of IoT technologies with the security measures necessary to protect sensitive medical data and ensure the reliability of connected medical devices. The Embedded Systems cluster, the blue cluster, examines the role of embedded systems in the design and operation of medical devices, highlighting innovations that enable more efficient and effective healthcare delivery. These clusters provide a comprehensive overview of the current state of research and emerging trends in the field of advanced medical technologies, offering valuable insights for researchers and practitioners alike.

## 5. AI and augmented reality in medical devices

The medical device industry is being transformed by Artificial Intelligence (AI) and Augmented Reality (AR) by increasing diagnostic accuracy, improving surgical precision, and providing immersive training environments. AI algorithms analyze vast amounts of medical data to aid in diagnosis, treatment planning, and patient monitoring, while AR provides real-time visualizations that assist surgeons during complex procedures and offer interactive educational tools for medical professionals.

The top authors in the field of AI and AR in medical devices, as presented in Table 2, are making significant contributions to advancing this innovative area of healthcare. Leading the list is Chen Y, who has an H-index of 6, emphasizing their impactful work on integrating augmented reality with artificial intelligence to develop diagnostic and therapeutic capabilities. Following closely is Cercenelli L, with an H-index of 3, focusing on using these technologies to improve surgical outcomes and medical training. Kamel B M, despite an H-index of 2, has a notable citation count of 247, reflecting significant contributions to the field. Other prominent authors such as Kleemann M and Laukkavirta M, although having lower H-indexes and citations, are actively engaged in exploring the applications of machine learning and computer vision in medical devices, further pushing the boundaries of what is possible with AI and AR in healthcare.

**Table 2.** Top authors in AI and AR in medical devices.

Rank	Author	H_index	TC
1	Chen Y	6	232
2	Cercenelli L	3	50
3	Kamel B M	2	247
4	Kleemann M	2	6
5	Laukkavirta M	1	1

### 5.1 Overview of AI and AR in medical devices

The integration of AI and AR in medical devices is transforming the healthcare landscape, enhancing efficiency, safety, and patient outcomes. Authors in ^28^ highlight that AI and AR advancements are improving diagnostics, personalized treatments, and patient monitoring. Ho and al. ^29^ discuss how AI enhances the precision of converting mechanical to electrical signals in medical devices, while AR provides real-time visual feedback, improving usability and efficiency. Nguyen and al. ^30^ explore the impact of AI and AR on healthcare within the digital economy, particularly in health metaverses, which increase patient engagement and treatment outcomes through virtual and augmented reality.

## 6. IoT and cybersecurity in medical devices

The integration of the Internet of Things (IoT) and the Internet of Medical Things (IoMT) with cybersecurity measures in medical devices is transforming healthcare by enhancing connectivity, real-time monitoring, and data security. These technologies improve patient care by enabling continuous monitoring and timely interventions while ensuring the security of sensitive health data against cyber threats.

In the rapidly evolving field of IoT and cybersecurity in medical devices, Table 3 shows that top authors such as Xu Y and Thirugnanam M are at the forefront of research, addressing significant challenges in securing interconnected medical devices. Their work focuses on enhancing data privacy and implementing robust cybersecurity measures to protect sensitive health information. Authors like Sharma S and Alizadehsani R are contributing valuable insights into the integration of IoT technologies with existing medical infrastructures, aiming to increase real-time monitoring and patient care. Feng X, although with a lower H-index, is also engaged in exploring innovative solutions for secure communication protocols within medical networks. Collectively, these authors are making significant contributions to ensuring that the benefits of IoT in healthcare are realized without compromising security and privacy.

**Table 3.** Top authors in IoT and Cybersecurity in medical devices.

Rank	Author	H_index	TC
1	Xu Y	4	127
2	Thirugnanam M	2	135
3	Sharma S	2	85
4	Alizadehsani R	2	29
5	Feng X	1	1

### 6.1 Overview of IoT and cybersecurity

The integration of IoT and cybersecurity in medical devices is revolutionizing healthcare by enhancing connectivity, real-time monitoring, and data security. N. M. Thomasian and al. ^11^ discuss the role of IoT in improving the quality and efficiency of medical services, emphasizing the importance of AI and machine learning in enhancing cybersecurity. Messinis and al. ^12^ explore security enhancements for IoMT, highlighting techniques to protect medical data and ensure patient safety. O. Pournik and al. ^13^ present a single message identification method for IoT, addressing the need for secure communication protocols in medical devices. Rocha and al. ^21^ review the use of edge AI for IoMT, discussing the benefits of real-time data processing and cybersecurity measures. Razdan and al. ^22^ provide an overview of emerging trends in IoMT, focusing on the cybersecurity challenges and solutions for protecting medical devices.

## 7. Embedded systems in medical devices

Embedded systems play a significant role in the development and functionality of modern medical devices, enhancing their reliability, efficiency, and real-time performance. These systems integrate hardware and software to perform specific functions, often within the constraints of real-time operations, making them vital for applications such as patient monitoring, diagnostic tools, and therapeutic devices.

The field of embedded systems in medical devices is critical for developing dependable, efficient, and real-time healthcare solutions. Leading this research, as mentioned in Table 4, is Lysecky R, whose work focuses on optimizing the hardware and software integration within medical devices. Adegbija T has also made significant advancements in microcontroller technologies and their applications in medical devices. Surrel G is notable for contributions to enhancing the real-time performance of these systems, which is vital for applications like patient monitoring and diagnostic tools. Furthermore, Nunes IO and Simalatsar A are engaged in collaborative efforts that push the boundaries of secure and efficient embedded systems, ensuring that these technologies can meet the stringent demands of the healthcare industry. These authors exemplify the innovative spirit and technical expertise driving progress in embedded systems for medical applications.

**Table 4.** Top authors in Embedded Systems in medical devices.

Rank	Author	H_index	TC
1	Lysecky R	3	22
2	Adegbija T	2	73
3	Surrel G	1	71
4	Nunes IO	1	19
5	Simalatsar A	1	6

### 7.1 Overview of Embedded Systems in medical devices

The integration of technology enhances the accuracy, safety, and efficiency of medical treatments and monitoring systems. Authors in ^31^ address memory safety challenges in life-critical medical devices with TrustFlow-X, a hardware/software co-designed framework ensuring fine-grained control-flow integrity against memory-based attacks. Similarly, authors in ^32^ develop a wearable, energy-efficient system for long-term obstructive sleep apnea (OSA) monitoring using a single-channel ECG signal, bridging home healthcare and professional supervision with real-time, patient-specific detection. Alena Simalatsar and al. introduce a method using Timed Automata extended with Tasks (TAT) for representing medical guidelines, addressing structural issues, enabling formal verification, and improving medical service protocols. ^33^

## 8. Discussion

This study has highlighted significant trends and developments in integrating advanced technologies in medical devices, focusing on AI, IoMT (Internet of Medical Things), AR (Augmented Reality), big data analytics, and cybersecurity. Our review of publications from 2010 to 2024 reveals a growing interest in these technologies, with notable contributions from Africa, particularly Morocco. The research identified three major clusters: AI and AR, IoT and cybersecurity, as well as embedded systems, each playing a critical role in modernizing healthcare. The findings align with existing literature that underscores the transformative potential of AI and IoMT in healthcare. AI is revolutionizing diagnostics and treatment planning, as noted by A. Abdaoui and al. ^18^ Similarly, IoMT and AR technologies enhance real-time monitoring and increase surgical precision. ^5^^,^^30^

AI, encompassing Machine Learning, Deep Learning, and Natural Language Processing, can analyze large datasets and provide predictive analytics to increase diagnostic accuracy and enable personalized treatment plans by identifying patterns in patient data that may be missed by human clinicians, leading to early intervention. ^19^^,^^20^ For example, AI has revolutionized medical imaging for conditions like cancer, ^18^ while NLP interprets unstructured data from clinical notes and Electronic Health Records (EHRs). ^1^ Additionally, AI combined with human-computer interaction (HCI) develops patient interfaces, making medical devices more user-friendly and efficient, while AI-driven simulations provide risk-free hands-on training for medical professionals. ^1^^,^^2^ AR technology has found numerous applications in medical training and surgical procedures. By overlaying digital information onto the physical world, AR can provide surgeons with real-time guidance, enhancing precision during complex surgeries and leading to better patient outcomes. ^34^ For instance, AR can display vital signs, anatomical landmarks, and surgical pathways directly onto the surgeon’s field of view, minimizing the need to look away from the patient to consult separate monitors. ^26^ This hands-free access to crucial data not only improves surgical accuracy but also reduces the likelihood of errors and shortens operation time. ^25^ IoMT’s connectivity enables continuous patient monitoring, which is crucial for managing chronic conditions and facilitating timely interventions. Devices such as wearable sensors, remote monitoring systems, and smart implants collect and transmit health data in real-time, allowing healthcare providers to monitor patients’ conditions continuously and adjust treatments as needed. ^21^ These technologies provide valuable insights into patients’ daily activities, vital signs, and overall health status, enabling proactive healthcare management. For example, wearable sensors can track parameters like heart rate, blood pressure, glucose levels, and physical activity, alerting both patients and healthcare providers to any abnormalities or significant changes. ^23^ This continuous flow of data allows for early detection of potential health issues, such as arrhythmias or spikes in blood sugar levels, enabling timely interventions that can prevent complications and hospitalizations.

However, with the increasing transmission of sensitive health data, data privacy and security have become paramount concerns. Robust cybersecurity measures are essential to protect against data breaches and unauthorized access, ensuring patient confidentiality and data integrity. ^12^ As IoMT devices collect and transmit vast amounts of personal health information, they become attractive targets for cyberattacks. Therefore, implementing advanced security protocols is critical to safeguarding this data. Embedded systems are crucial for modern medical devices, providing necessary computational power for specific applications like medical imaging, patient monitoring, and wearable health devices. They refine functionality, reliability, and precision. In medical imaging, they process data in real-time for high-resolution images, aiding diagnosis and treatment. In patient monitoring, they track vital signs continuously, enabling timely interventions. Wearable devices use embedded systems to collect and analyze health data, offering actionable insights to users and healthcare providers. ^31^^–^^33^

Despite advancements, significant challenges remain. Infrastructure limitations, such as the need for reliable internet connectivity and advanced computational resources, hinder the widespread adoption of these technologies, especially in rural or underserved areas. This digital divide can result in disparities in healthcare quality. Additionally, interoperability issues between various devices and systems complicate integration into existing healthcare frameworks, as different manufacturers often use proprietary technologies and standards. Developing standardized protocols and interfaces is crucial for seamless communication and integration across devices from different manufacturers.

Industry 4.0, characterized by the integration of cyber-physical systems, IoT, and cloud computing, has the potential to significantly impact medical devices powered by advanced technologies. These innovations enable smart manufacturing, enhancing the production and scalability of medical devices. AI-driven analytics can develop quality control by identifying defects early, ensuring high standards of safety and efficacy. ^1^^,^^21^ IoT-enabled devices facilitate real-time monitoring of manufacturing conditions, allowing for adjustments to maintain consistency and regulatory compliance. ^6^ Moreover, AR can be used for remote assistance and training, providing real-time visual instructions and guidance, enhancing efficiency and expertise in manufacturing. ^24^^,^^27^ The integration of technologies like 3D printing with AI and IoT allows for personalized manufacturing of medical devices, such as custom-fitted prosthetics and implants, significantly advancing the field and improving patient outcomes.

## 9. Conclusion

This article has explored the transformative impact of advanced technologies such as Artificial Intelligence (AI), the Internet of Medical Things (IoMT), augmented reality (AR), big data analytics, and cybersecurity on the medical device industry. A comprehensive analysis of academic publications from 2010 to 2024 using data from Scopus and Web of Science identified significant trends, key contributors, and emerging themes in these fields. The analysis of trend keywords reveals a growing interest in “Machine Learning,” “Artificial Intelligence,” “Internet of Things,” “Medical Devices,” and “Cybersecurity.” These keywords highlight the areas of focus and innovation in the medical device industry, reflecting the increasing adoption of advanced technologies to improve healthcare outcomes.

Africa’s position in this technological landscape is emerging, with notable contributions from countries such as Egypt, South Africa, and Morocco. Morocco is making significant strides in advancing healthcare technologies, demonstrating a growing commitment to scientific research and development in this field. These efforts are fostering innovation and placing African nations on the global map of technological advancements in healthcare.

The research identified three major clusters: AI and AR, IoT and cybersecurity, and embedded systems. The AI and AR cluster focuses on integrating these technologies to boost up diagnostic accuracy and patient outcomes. The IoT and cybersecurity cluster emphasizes the importance of secure and reliable interconnected medical devices. The embedded systems cluster highlights innovations in hardware and software integration, which are critical for real-time operations and the efficiency of medical devices. These clusters underscore the multifaceted approach required to advance medical technologies and address the challenges of modern healthcare.

### Future directions

Our future perspective will concentrate on studying the integration and impact of advanced technologies within a single medical device. This focused approach will allow us to deeply investigate how specific technologies like AI, IoMT, and AR can be used to develop the functionality, safety, and efficiency of a particular medical device. By focusing on one device, we aim to provide detailed insights into the technical challenges, potential improvements, and practical applications of these technologies in a healthcare setting. This will not only contribute to the body of knowledge but also pave the way for targeted innovations that could revolutionize the design and use of medical devices.

Key areas of exploration include the benefits of AI in enhancing device functionality by learning from vast data and making real-time decisions, improving diagnostic accuracy and patient outcomes, and aiding in predictive maintenance. IoMT integration will be studied for its potential to create a network of connected devices, allowing continuous monitoring and real-time data analysis, as exemplified by smart insulin pumps. AR technology’s ability to increase surgical precision and medical training will be evaluated. Research on cybersecurity measures will focus on protecting sensitive health data transmitted by IoMT devices. Collaborative research with healthcare professionals, patients, and industry stakeholders will ensure the development of user-friendly, technologically advanced solutions aligned with healthcare sector requirements.

#### Ethics and consent

All ethical guidelines were strictly adhered to during the conduct of this research.

## Data Availability

Figshare: Comprehensive Dataset of Academic Publications on the Integration of Advanced Technologies in Medical Devices with PRISMA Documentation (2010-2024), https://doi.org/10.6084/m9.figshare.26412223.v2. ^35^ Data are available under the terms of the Creative Commons Attribution 4.0 International license (CC-BY 4.0).
