# Developing medical devices with emerging technologies: trends, challenges, and future directions

**DOI:** 10.12688/f1000research.154869.3

**Published:** 2026-08-17

**Authors:** Achraf akkaoui, Yassine ZAHIDI, Mohamed El Moufid, Wafaa DACHRY, Hassan GZIRI, Hicham Medromi

**Affiliations:** 1 Engineering, Industrial Management and Innovation (EIMI), Hassan 1st University Faculty of Science and Technology Settat, Settat, Chaouia-Ouardigha, Morocco; 2 Foundation for Research Development and Innovation in Science and Engineering, Casablanca, Grand Casablanca, Morocco; 3 The International Academy of Scientific Francophone (IASF), Rabat, Morocco

**Keywords:** Artificial Intelligence, Internet of Medical Things, Augmented Reality, Cybersecurity, Medical Devices, Health care, Embedded systems

## Abstract

This study presents a bibliometric analysis of the rapid advancement and integration of emerging technologies in medical device development, focusing on Artificial Intelligence (AI), the Internet of Medical Things (IoMT), Augmented Reality (AR), and cybersecurity. Using data from the Scopus and Web of Science databases, we analyzed 3,094 publications from 2010 to 2024 to map trends, challenges, and future directions in the field. The analysis shows substantial progress in patient care through AI and IoMT, which enable predictive analytics, personalized treatment planning, and real-time monitoring; AR is reshaping medical training and surgical precision; and cybersecurity has become critical to protecting sensitive health data. Persistent challenges include data-privacy concerns, infrastructure limitations, and interoperability issues. We also examine Africa’s contributions, with particular emphasis on Morocco’s emerging role. Three major research clusters are identified: AI and AR, IoT and cybersecurity, and embedded systems. Beyond the bibliometric mapping, we provide a comparative evaluation of the technologies, representative case studies of advanced medical devices, an expanded treatment of cybersecurity for AI-enabled devices, and a dedicated discussion of ethical, legal, and regulatory considerations. As a bibliometric analysis, the study characterizes research activity and does not perform critical appraisal or risk-of-bias assessment of individual studies. The work offers a foundation for further research and innovation in this rapidly evolving field.

## 1. Introduction

The rapid advancement of technology has significantly impacted the medical field, introducing innovative solutions that enhance patient care. Among these technologies, Artificial Intelligence (AI) and the Internet of Medical Things (IoMT) stand out for their transformative potential. AI, through machine learning, deep learning, natural language processing, and data analysis, is changing how medical data are interpreted and used, enabling predictive analytics and personalized treatment plans that contribute to more accurate diagnoses and improved patient outcomes. ^1– 4^ IoMT connects medical devices through the internet, enabling real-time data collection, monitoring, and analysis, thereby improving the efficiency and accuracy of healthcare delivery. ^5– 9^

Integrating these technologies into medical devices raises challenges, notably data privacy and security. These are paramount given the sensitivity of health information, and robust cybersecurity is required to protect against breaches and unauthorized access. ^10– 12^ Infrastructure limitations, reliable connectivity and advanced computational resources, pose further barriers, while interoperability issues between devices and systems complicate integration into existing healthcare frameworks. ^13, 14^ Despite these barriers, the potential benefits of AI, IoMT, AR, big data analytics, and cybersecurity are driving substantial research and development.

This study examines the evolution and current landscape of advanced technologies in the medical field by analyzing academic publications from 2010 to 2024, using data from two prominent databases, Scopus and Web of Science, selected for their extensive coverage of peer-reviewed literature in science, technology, and medicine. The methodology is a systematic keyword-based bibliometric analysis, merging datasets, removing duplicates with the Bibliometrix library in R, and analyzing and visualizing the data with Biblioshiny and VOSviewer. ^15^

Existing bibliometric and review studies on emerging technologies in healthcare typically examine a single technology in isolation, for example AI, IoMT, or AR individually. Comparatively few studies map the convergence of AI, IoMT, AR/VR, embedded systems, and cybersecurity as an integrated medical-device ecosystem, and fewer still foreground the contribution of African research systems. This study addresses that gap by jointly analyzing these technologies across a large two-database corpus and by explicitly situating Africa’s, and particularly Morocco’s, emerging role within the global landscape. This integrated, region-aware perspective is the principal novelty and contribution of the present work relative to prior reviews.

This article seeks to answer the following questions regarding the integration of advanced technologies in medical devices:

RQ1: What are the contributions and advancements made by different regions, particularly Africa, in this technological landscape?

RQ2: What are the current trends and advancements in the integration of advanced technologies in medical devices?

RQ3: What are the major challenges and barriers to the implementation of these advanced technologies in the medical field?

The remainder of the paper is organized as follows: Section 2 describes the methodology and materials; Section 3 gives an overview of the advanced technologies covered (AI, IoMT, AR, big data analytics, and cybersecurity); Section 4 presents the bibliometric findings and trends; Sections 5–7 examine the three research clusters; Section 8 discusses the implications, a comparative evaluation, case studies, and cybersecurity for AI-enabled devices; Section 9 addresses ethical, legal, and regulatory considerations; and Section 10 concludes with future research directions.

## 2. Methods

This work is a bibliometric analysis of academic publications on advanced medical technologies, principally AI and IoMT and their convergence with AR, embedded systems, and cybersecurity. A bibliometric analysis characterizes the structure, volume, and dynamics of a research field through publication metadata (authors, sources, keywords, citations, and collaboration). It is therefore distinct from a systematic review: it does not register a protocol, extract clinical outcome data, or perform critical appraisal/risk-of-bias assessment of individual studies. We adopted the PRISMA 2020 flow ^16^ only to make the search and selection process transparent and reproducible, not to imply evidence synthesis. Data were drawn from Scopus and Web of Science, selected for their broad coverage of peer-reviewed science, technology, and medicine literature.

### 2.1. Search strategy

Both databases were searched on May 2024 for documents published between 2010 and 2024. The 2010 lower bound was chosen because the technological advances since 2010 have transformed the medical-device landscape. The database-specific queries were as follows.

We initiated our data collection with a search using the query: ((“medical devices” AND (“artificial intelligence” OR “internet of medical things” OR “deep learning” OR “Internet of Things” OR “machine learning” OR “Augmented reality”)). we refined our dataset by applying inclusion criteria based on specific keywords such as “Artificial Intelligence” or “Internet Of Things” or “Medical Devices” or “Health Care” or “Machine Learning” or “Biomedical Equipment” or “Big Data” or “Medical Device” or “Deep learning” or “Wearable Devices” or “Internet Of Medical Things” or “Cloud Computing” or “Augmented Reality” or “Wearable Medical Devices” or “Virtual Reality” or “Real-time”. This refinement was geared towards focusing on the specific technologies and their applications in medical devices.

### 2.2. Inclusion and exclusion criteria

Publications were included if they (1) addressed the integration of AI, IoMT, AR, cybersecurity, or related emerging technologies in medical devices; (2) were peer-reviewed journal articles, conference papers, reviews, or book chapters; and (3) were available in English with complete bibliographic metadata. Records were excluded if they were unrelated to medical devices, were commentaries, editorials, or non-scientific reports, or lacked sufficient metadata for bibliometric processing.

### 2.3. Screening, de-duplication, and selection

Records retrieved from the two databases were exported and combined in R using the Bibliometrix package, ^17^ which merged the datasets and removed duplicates by matching on Digital Object Identifier (DOI) and, where DOIs were absent, on normalized title and source. Titles, abstracts, and keywords were then screened against the inclusion/exclusion criteria. Screening was performed independently by the authors of this manuscript; as well as the disagreements were resolved by them.

**Figure 1.**
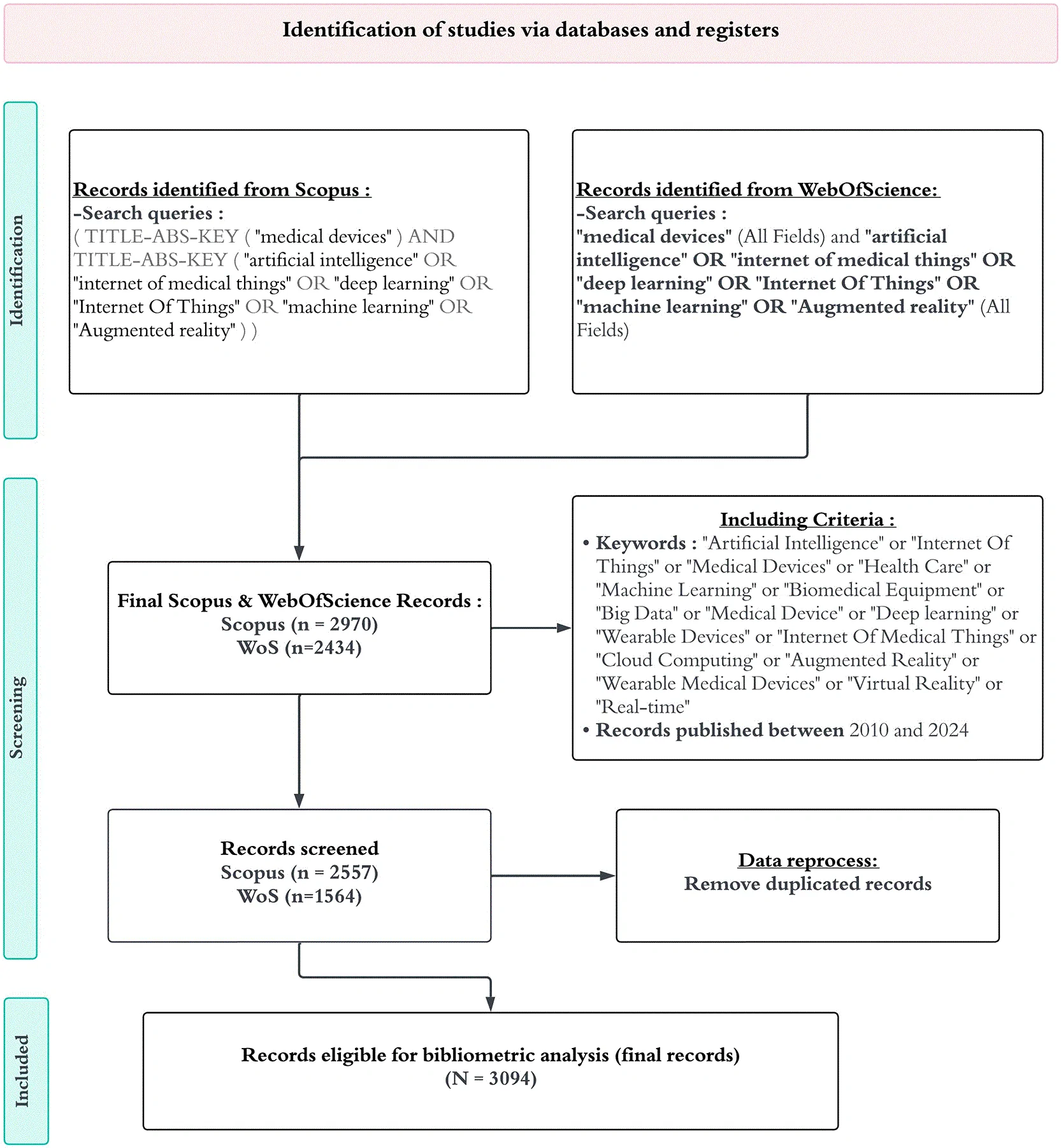
PRISMA diagram for data collection and refinements.

The selection process is summarized in the PRISMA flow diagram ^18^ ( Figure 1). The number of records identified, de-duplicated, screened, excluded, and finally included is reported there, yielding a final corpus of 3,094 documents.

### 2.4. Analysis and visualization tools

For advanced analysis and visualization, we used Biblioshiny from Bibliometrix, ^17^ and VOSviewer, ^15^ the latter constructing bibliometric maps with the VOS (Visualization of Similarities) technique, which uses mathematical algorithms to represent relationships between items.

### 2.5. Bias and quality considerations

Two forms of limitation are relevant. First, publication bias may arise from database-coverage differences and the predominance of English-language publications; to mitigate this we combined Scopus and Web of Science to broaden geographical and disciplinary representation, while acknowledging that indexing practices and language restrictions may still underrepresent emerging regions and local scientific outputs. Second, and by design, a bibliometric analysis of publication metadata does not assess the methodological quality, internal validity, or clinical validity of the individual studies it counts; consequently, no formal critical appraisal or risk-of-bias assessment was performed. This is an inherent limitation of the design rather than an omission, and readers should interpret the findings as a map of research activity rather than a synthesis of clinical evidence.

Table 1 summarizes the bibliometric profile of the corpus (2010–2024): 3,094 documents from 1,747 sources, with a 27.8% annual growth rate, an average of 13.92 citations per document, and a mean document age of 3.24 years. The dataset includes 10,695 authors, of whom a substantial share participates in international collaboration (16.29%). Articles predominate (1,405), followed by conference papers (580), reviews (376), and book chapters (165).

**Table 1.** Main information of final records.

Description	Results
MAIN INFORMATION ABOUT DATA	
Timespan	2010:2024
Sources (Journals, Books, etc.)	1747
Documents	3094
Annual Growth Rate %	27,8
Document Average Age	3,24
Average citations per doc	13,92
References	115522
DOCUMENT CONTENTS	
Keywords Plus (ID)	11335
Author's Keywords (DE)	7636
AUTHORS	
Authors	10695
Authors of single-authored docs	216
AUTHORS COLLABORATION	
Single-authored docs	231
Co-Authors per Doc	4,74
International co-authorships %	16,29
DOCUMENT TYPES	
Article	1405
Book chapter	165
Conference paper	580
Review	376

## 3. Advanced technologies overview

### 3.1. Artificial intelligence

Artificial Intelligence (AI) simulates human intelligence in programmed machines capable of learning and reasoning. It includes machine learning, where algorithms improve from data without explicit programming; deep learning, which processes data through neural networks; natural language processing (NLP), enabling machines to interpret human language; and computer vision, which interprets visual data. In healthcare, AI applications range from robot-assisted surgery offering high precision to virtual nursing assistants that reduce unnecessary hospital visits and staff burden. ^19– 21^ AI’s impact extends across sectors, for example, real-time fraud detection in finance and autonomous-vehicle safety in transportation, illustrating its broad transformative potential. ^1, 2^

### 3.2. Internet of things and internet of medical things

The Internet of Things (IoT) links physical objects embedded with sensors and software that connect and exchange data over the internet, enabling real-time monitoring, control, and optimization across sectors; in manufacturing, for instance, IoT supports predictive maintenance that reduces downtime. ^5, 6, 12^

In healthcare, the Internet of Medical Things (IoMT) applies IoT to patient monitoring and care: smart inhalers and wearable trackers collect health data and improve chronic-disease management, and IoMT supports remote patient monitoring that enables informed, timely clinical decisions, moving healthcare toward more patient-centred, data-driven delivery. ^7– 9, 22– 24^

### 3.3. Augmented reality and virtual reality

Augmented Reality (AR) enhances the physical world with digital overlays, providing real-time information; in medicine, AR can display patient data during procedures for more precise and safer surgery, and in education it brings complex concepts to life. Virtual Reality (VR) creates immersive environments widely used in therapy and rehabilitation, for example, controlled, intensive sessions for patients recovering from stroke or injury, accelerating recovery without the physical constraints of the real world. ^14, 25– 29^

### 3.4. Big data analysis

Big-data analysis examines vast datasets to uncover patterns, trends, and insights. In healthcare it is used to analyze patient information, predict disease patterns, and anticipate treatment outcomes, supporting more effective, individualized interventions. Its growing capabilities continue to influence societal efficiency and economic activity. ^3, 4^

### 3.5. Cybersecurity

As digital technologies permeate all aspects of life, cybersecurity has become a critical pillar of the digital economy, essential for protecting sensitive data and preventing cyber threats. In healthcare, robust cybersecurity protects patient records and ensures the integrity of medical devices. The growing adoption of IoT devices has expanded the attack surface, requiring advanced strategies to secure interconnected devices and networks; emerging approaches include the use of AI to detect anomalous patterns quickly and accurately, enabling proactive threat prevention. ^10– 12^

## 4. Analysis of collected data

The integration of advanced technologies in medical devices and healthcare systems has significantly transformed patient care, diagnostics, and treatment. The following sections examine keyword trends, global contributions, research clusters, and collaboration and growth dynamics.

### 4.1. Words’s frequency over time

**Figure 2.**
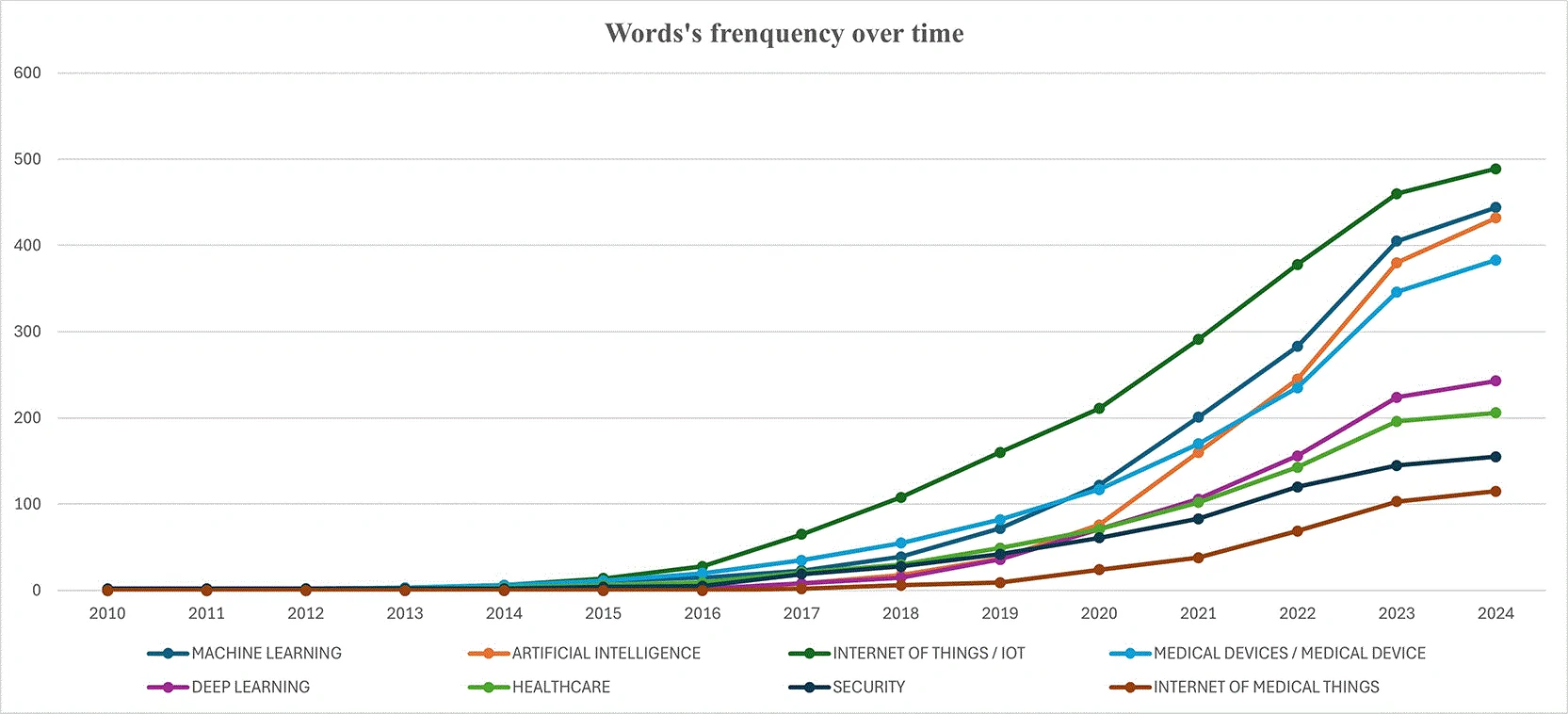
The frequency of various keywords overtime.

Figure 2 illustrates the frequency of key terms related to advanced technologies in healthcare over 2010–2024. Terms such as “Machine Learning”, “Artificial Intelligence”, “Deep Learning”, “Healthcare”, “Security”, “Internet of Things (IoT)”, “Medical Devices”, and “Internet of Medical Things” rise markedly, particularly from 2016 onward. These trends have identifiable drivers. The steep rise of “Deep Learning” and “Artificial Intelligence” from around 2015–2016 coincides with the breakthrough performance of deep neural networks in computer vision and their rapid diffusion into medical imaging. The acceleration of “Healthcare”, “Internet of Things”, and “Internet of Medical Things” around 2019–2021 aligns with the expansion of remote patient monitoring and telehealth, which the COVID-19 period intensified. The consistent growth of “Security” reflects rising attention to the protection of sensitive medical data following high-profile healthcare and device-related cyber-incidents. Together these trends indicate not merely increased usage but a field reorganizing around connected, data-driven, and AI-enabled devices.

### 4.2. Countries’ scientific production

**Figure 3.**
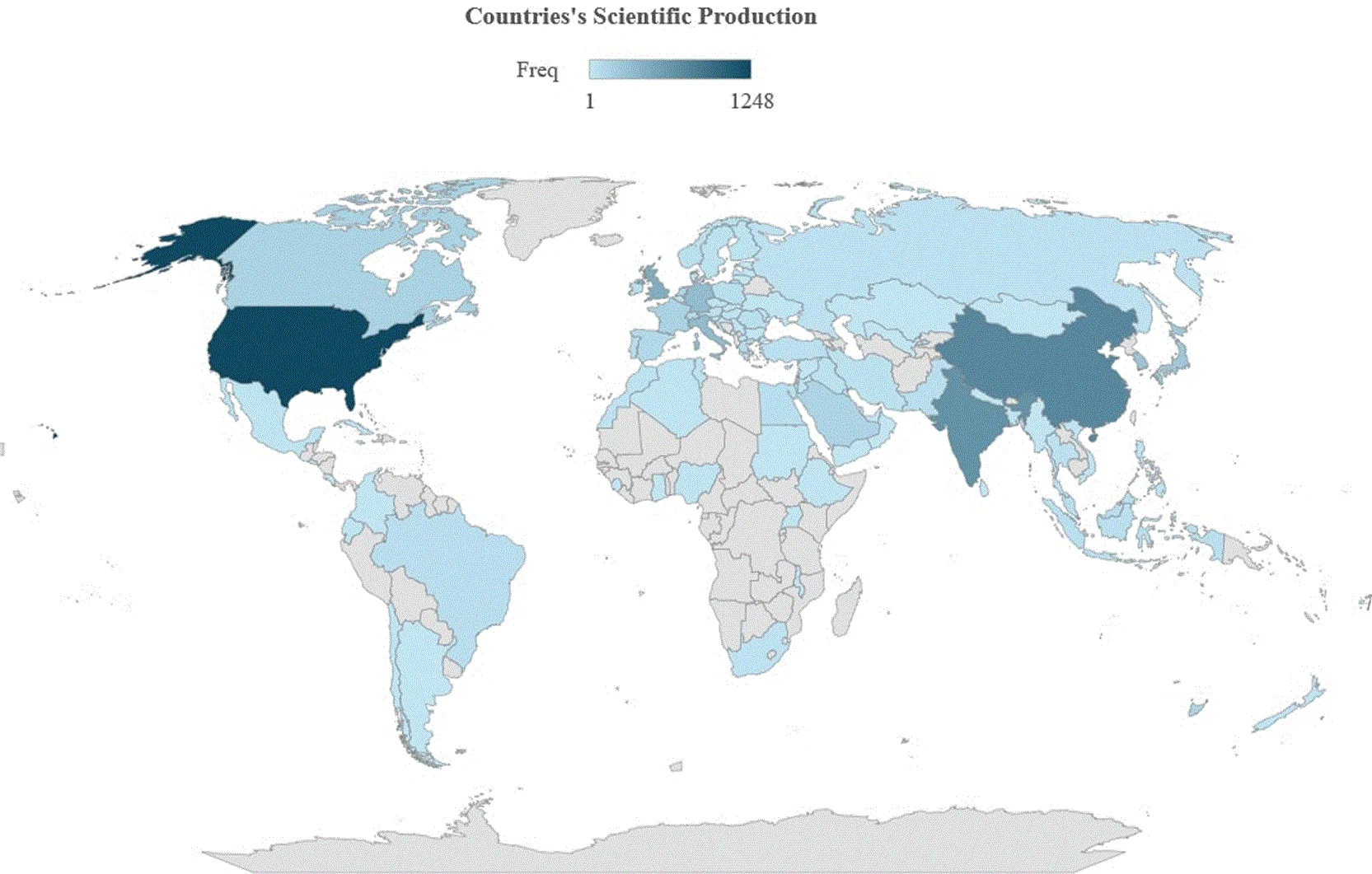
Countries' scientific production of advanced technologies for medical devices.

Figure 3 shows the global distribution of scientific production. The United States leads (1,248 publications), followed by China (737), India (654), and the United Kingdom (437); other notable contributors include Italy, Germany, South Korea, and Japan. Africa’s output, while smaller, shows emerging contributions from Egypt (54), South Africa (22), and Morocco (22), with Morocco notable for its growing role on the continent.

These are raw publication counts and should be read with care, because absolute counts favor large, well-funded research systems. The leading positions of the United States, China, and India are consistent with their population size, aggregate R&D investment, and established biomedical-device industries, rather than indicating higher per-researcher productivity. Normalizing output by population or by gross domestic expenditure on R&D would reorder the ranking and reveal high per-capita performers among smaller research systems; several European countries, for example, produce large volumes relative to population. We therefore present the counts as indicators of aggregate research capacity, and we identify normalized bibliometric benchmarking (per capita, per GDP, or per R&D expenditure) as an important direction for future work. Read in this light, the African figures represent a genuine and growing, if still emerging, participation rather than a marginal one.

### 4.3. Keyword clusters analysis

**Figure 4.**
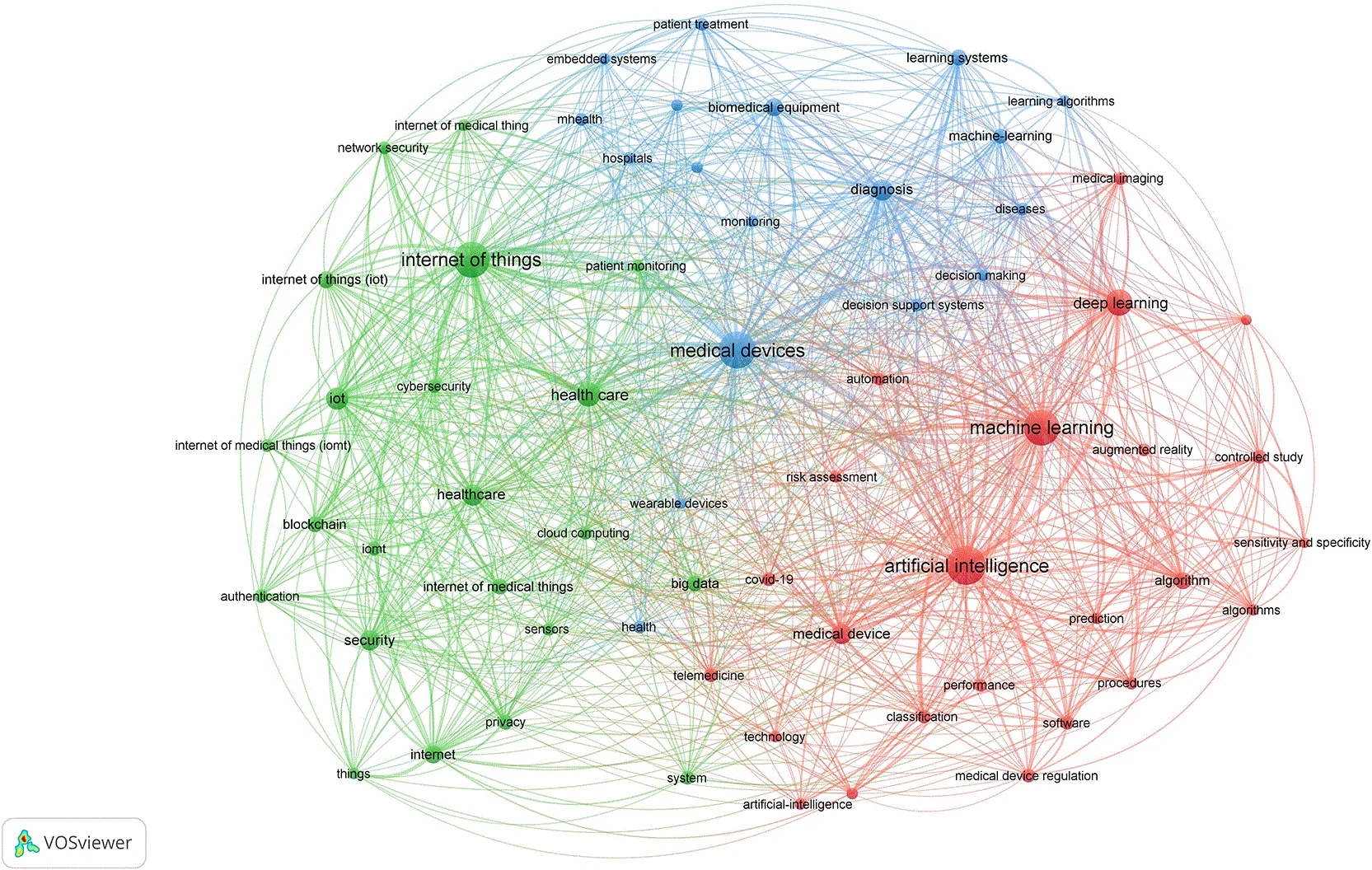
Visualization of key research clusters in advanced medical technologies using VOSviewer.

VOSviewer ^15^ was used to map co-occurrence structure with a minimum keyword co-occurrence of 50, yielding 66 keywords and three clusters Figure 4: (i) an AI and AR cluster (red), on integrating AI and AR to improve diagnostic accuracy and patient outcomes; (ii) an IoT and cybersecurity cluster (green), on protecting sensitive medical data and ensuring the reliability of connected devices; and (iii) an embedded systems cluster (blue), on hardware/software integration for efficient, real-time device operation.

Two caveats qualify this map. First, the clusters are not static: the AI/AR cluster expanded most rapidly in the second half of the period, and cybersecurity terms migrated from the periphery toward the core as connectivity increased, indicating a field consolidating around AI-enabled, connected devices. Second, the high minimum co-occurrence threshold (50) biases the map toward high-frequency, generic terms and can suppress specialized but important topics; notably, interoperability, standardization, and post-market surveillance are comparatively under-represented relative to their practical significance. The three-cluster structure should therefore be read as a robust first-order summary rather than an exhaustive taxonomy, and the apparent tidiness of three clusters is partly an artefact of the threshold choice.

### 4.4. Collaboration patterns and growth trajectory

The corpus also carries a clear collaboration and growth signal. Co-authorship averages 4.74 authors per document, with 16.29% of documents involving international collaboration and only 231 single-authored documents out of 3,094, indicating a strongly team-based, increasingly cross-border research culture, consistent with the multidisciplinary nature of medical-device engineering (spanning clinical, computational, and hardware expertise). The 27.8% compound annual growth rate over 2010–2024 substantially outpaces the growth of the general biomedical literature, quantitatively substantiating the “emerging” characterization of the field rather than asserting it qualitatively. Read together, the rising international co-authorship and the steep growth trajectory suggest a field transitioning from isolated national efforts toward a globally networked research enterprise.

## 5. AI and augmented reality in medical devices

AI and AR are transforming the medical-device industry by increasing diagnostic accuracy, improving surgical precision, and providing immersive training environments. AI algorithms analyze large volumes of medical data to support diagnosis, treatment planning, and monitoring, while AR provides real-time visualizations that assist surgeons during complex procedures and offer interactive educational tools.

Table 2 lists the top authors in AI and AR in medical devices. Chen Y leads (H-index 6) with work integrating AR and AI for diagnostic and therapeutic capabilities, followed by Cercenelli L (H-index 3) on surgical outcomes and training. Kamel B M, despite a lower H-index (2), has a notable citation count (247). Kleemann M and Laukkavirta M are active in machine-learning and computer-vision applications for medical devices.

**Table 2.** Top authors in AI and AR in medical devices.

Rank	Author	H_index	TC
1	Chen Y	6	232
2	Cercenelli L	3	50
3	Kamel B M	2	247
4	Kleemann M	2	6
5	Laukkavirta M	1	1

### 5.1. Overview of AI and AR in medical devices

The integration of AI and AR is enhancing efficiency, safety, and patient outcomes. Advances in AI and AR are improving diagnostics, personalized treatment, and monitoring ^29^; AI enhances the precision of converting mechanical to electrical signals in devices, while AR provides real-time visual feedback that improves usability ^30^; and AI and AR are increasingly applied in health metaverses that raise patient engagement and treatment outcomes through virtual and augmented experiences. ^31^

## 6. IoT and cybersecurity in medical devices

Integrating IoT and IoMT with cybersecurity is transforming healthcare by enhancing connectivity, real-time monitoring, and data security, improving care through continuous monitoring and timely intervention while protecting sensitive health data against cyber threats.

Table 3 shows leading authors in this area. Xu Y and Thirugnanam M address the security of interconnected medical devices and data privacy; Sharma S and Alizadehsani R contribute to integrating IoT with medical infrastructures for real-time monitoring; and Feng X explores secure communication protocols within medical networks.

**Table 3.** Top authors in IoT and Cybersecurity in medical devices.

Rank	Author	H_index	TC
1	Xu Y	4	127
2	Thirugnanam M	2	135
3	Sharma S	2	85
4	Alizadehsani R	2	29
5	Feng X	1	1

### 6.1. Overview of IoT and cybersecurity

IoT and cybersecurity are jointly reshaping healthcare. IoT improves the quality and efficiency of medical services, with AI and machine learning strengthening cybersecurity ^11^; security enhancements for IoMT protect medical data and patient safety ^12^; secure communication protocols address message-level identification needs ^13^; edge AI enables real-time data processing with embedded security ^22^; 21 and overviews of IoMT emphasize the cybersecurity challenges and solutions for protecting connected devices. ^23^

## 7. Embedded systems in medical devices

Embedded systems are central to modern medical devices, integrating hardware and software to perform specific functions under real-time constraints, for patient monitoring, diagnostics, and therapy, and enhancing reliability, efficiency, and precision.

Table 4 lists leading authors. Lysecky R focuses on hardware/software integration; Adegbija T advances microcontroller technologies; Surrel G improves real-time performance for monitoring and diagnostics; and Nunes IO and Simalatsar A work on secure, efficient embedded systems meeting the demands of healthcare.

**Table 4.** Top authors in Embedded Systems in medical devices.

Rank	Author	H_index	TC
1	Lysecky R	3	22
2	Adegbija T	2	73
3	Surrel G	1	71
4	Nunes IO	1	19
5	Simalatsar A	1	6

### 7.1. Overview of Embedded Systems in medical devices

Embedded integration enhances the accuracy, safety, and efficiency of treatment and monitoring. TrustFlow-X provides fine-grained control-flow integrity against memory-based attacks in life-critical devices ^32^; a wearable, energy-efficient single-channel ECG system enables long-term obstructive sleep apnea monitoring with real-time, patient-specific detection ^33^; and Timed Automata extended with Tasks (TAT) formalize medical guidelines to enable verification and improve protocols. ^34^

## 8. Discussion

This bibliometric analysis of publications from 2010 to 2024 reveals a growing and increasingly integrated research effort around AI, IoMT, AR, big-data analytics, and cybersecurity in medical devices, with three consolidating clusters (AI and AR; IoT and cybersecurity; embedded systems) and notable, emerging contributions from Africa, particularly Morocco. Beyond the volume of activity, the more consequential finding is qualitative: the medical device is shifting from a stand-alone instrument toward a connected, software-defined, and increasingly AI-enabled system, in which diagnostic intelligence, real-time connectivity, and security are co-designed rather than added after the fact.

### 8.1. Comparative evaluation of the technologies

The five technology families differ substantially in maturity, benefit, and implementation difficulty. Table 5 summarizes a comparative evaluation, which the surrounding discussion expands. AI/ML is the most transformative but faces the steepest validation, transparency, and data-governance hurdles; IoMT delivers immediate monitoring value but concentrates security and interoperability risk; AR/VR is clinically compelling in training and navigation yet remains comparatively niche and hardware-dependent; embedded systems are foundational and mature but constrained by real-time and safety-certification demands; and cybersecurity is a cross-cutting enabler whose absence undermines all of the others.

**Table 5.** Comparative evaluation of emerging technologies in medical devices.

Technology	Maturity	Key strengths	Implementation challenges	Clinical readiness
AI/ML	Rapidly maturing	Diagnostics, prediction, personalization	Validation, transparency/explainability, data bias, regulation	Growing (some cleared devices)
IoMT	Established, expanding	Continuous & remote monitoring	Security, interoperability, connectivity	High for monitoring
AR/VR	Emerging	Surgical navigation, training	Hardware cost, ergonomics, evidence base	Moderate/selective
Big data	Maturing	Population insight, pattern discovery	Data quality, integration, governance	Indirect/infrastructural
Embedded systems	Mature	Real-time, reliable device control	Real-time constraints, safety certification	High (foundational)
Cybersecurity	Maturing, urgent	Data protection, device integrity	Legacy devices, evolving threats, cost	Cross-cutting enabler

### 8.2. Representative case studies

Several devices deployed illustrate the practical impact of these technologies. Autonomous AI diagnosis: IDx-DR became the first FDA-authorized system to make an autonomous diagnostic decision without physician interpretation, detecting diabetic retinopathy from retinal images in primary-care settings. ^35^ AI-enabled cardiac monitoring: large-scale, app-based screening using photoplethysmography and single-lead ECG on consumer wearables has demonstrated feasibility for detecting atrial fibrillation at population scale. ^36^ Closed-loop insulin delivery: “artificial-pancreas” systems couple continuous glucose monitoring with algorithmic control of insulin pumps, exemplifying tight IoMT – embedded – AI integration in a life-critical device. Robot-assisted surgery and AR navigation: surgical robots combined with AR overlays of anatomy and imaging enhance precision and reduce the need to divert attention to separate monitors. ^27, 37^ These examples ground the bibliometric trends in concrete clinical value while also surfacing the validation, cost, and integration challenges discussed above.

### 8.3. Cybersecurity for AI-enabled medical devices

As devices become connected and AI-driven, the threat surface broadens beyond classical data breaches. Generative AI introduces new risks, training-data leakage, model-inversion and membership-inference attacks that can expose patient data, and prompt-injection against clinical language interfaces. Adversarial attacks can perturb medical images or signals to cause confident misclassification by diagnostic models, a demonstrated vulnerability with direct safety implications. ^38^ At the network level, distributed denial-of-service (DDoS) attacks can disrupt hospital connectivity and the availability of monitoring services, while man-in-the-middle (MITM) attacks can intercept or alter device telemetry between sensors, gateways, and clinical systems.

Correspondingly, privacy-preserving and secure-AI approaches are becoming central to intelligent healthcare systems. Federated learning trains models across institutions without moving raw patient data, reducing exposure while enabling multi-site learning ^39^; differential privacy adds calibrated noise to bound the information any individual record contributes to a model; and edge computing keeps sensitive processing on or near the device, lowering latency and limiting data in transit. ^22^ Combined with strong authentication, encryption, and secure-by-design development, these techniques form a layered defence appropriate to AI-enabled devices.

### 8.4. Implementation challenges and limitations of the study

Significant implementation challenges remain. Infrastructure limitations, reliable connectivity and computational resources, hinder adoption, especially in rural or underserved areas, risking a digital divide in care quality. Interoperability is constrained by proprietary standards, making standardized protocols and interfaces essential for seamless integration across manufacturers. Clinical adoption further depends on rigorous validation, workflow integration, clinician training, and trust in opaque AI models, where biased training data can produce unequal performance across populations and over-reliance on automation must be avoided.

This study has limitations beyond those inherent to its design (Section 2.5). The corpus is restricted to Scopus- and Web of Science-indexed, English-language documents, which may exclude relevant regional or non-English research; predefined keywords may omit studies using different terminology; and, as a bibliometric analysis, it maps research activity through metadata and does not assess the methodological or clinical quality of individual studies. Despite these limits, the results consistently indicate a shift toward more intelligent, connected, and data-driven medical devices.

## 9. Ethical, legal, and regulatory considerations

The integration of AI, IoMT, AR, and cybersecurity into medical devices raises ethical, legal, and regulatory issues that are inseparable from technical design. AI governance is consolidating: the European Union’s Artificial Intelligence Act establishes a risk-based framework that places most medical AI in a high-risk category with obligations for data quality, transparency, human oversight, and post-market monitoring, ^40^ while the United States Food and Drug Administration has advanced a framework for AI/ML-based Software as a Medical Device, including a predetermined change-control approach to safely manage model updates. ^41^

Medical-device regulation and software-lifecycle/security standards provide the compliance backbone. In the EU, the Medical Device Regulation (EU) 2017/745 governs safety and performance across the device lifecycle ^42^; IEC 62304 defines software life-cycle processes for medical-device software ^43^; and IEC 81001–5-1 specify security activities for health-software development. ^44^ These standards translate broad principles of safety and security into auditable engineering practice.

Patient-data privacy regimes complete the picture: the EU General Data Protection Regulation (GDPR) sets requirements for lawful processing, consent, and data-subject rights ^45^; the US Health Insurance Portability and Accountability Act (HIPAA) governs protected health information; and, in Morocco, Law 09–08 and its supervisory authority (CNDP) regulate the processing of personal data. Aligning device design with these overlapping regimes, governance, device compliance, and privacy, is essential to the credibility, safety, and lawful deployment of intelligent medical devices, and it is an area where interdisciplinary collaboration between engineers, clinicians, and legal experts is increasingly required.

## 10. Conclusion

This bibliometric analysis of academic publications from 2010 to 2024 characterized the integration of AI, IoMT, AR, big-data analytics, and cybersecurity in medical devices, identifying key trends, contributors, and three consolidating research clusters, AI and AR, IoT and cybersecurity, and embedded systems. Trend keywords such as “Machine Learning”, “Artificial Intelligence”, “Internet of Things”, “Medical Devices”, and “Cybersecurity” reflect the field’s reorganization around intelligent, connected devices.

Africa’s position is emerging, with notable contributions from Egypt, South Africa, and Morocco; Morocco in particular shows a growing commitment to research and development in this domain, helping to place African research on the global map. Beyond the mapping, the comparative evaluation, case studies, expanded cybersecurity analysis, and the treatment of ethical, legal, and regulatory considerations indicate that realizing the benefits of these technologies will require rigorous validation, robust security-by-design, interoperable standards, and clear governance. Continued interdisciplinary research, combined with policy support and infrastructure development, will be important to fully realize the benefits of these innovations on a global scale.

## Future directions

Our future work will concentrate on the integration and impact of advanced technologies within a single medical device. This focused approach will allow us to investigate in depth how specific technologies – AI, IoMT, and AR – can improve the functionality, safety, and efficiency of a particular device, providing detailed insight into the technical challenges, potential improvements, and practical applications in a real healthcare setting, and paving the way for targeted innovations in device design and use.

Key areas of exploration include: the role of AI in enhancing device functionality through learning from data, real-time decision-making, improved diagnostic accuracy, and predictive maintenance; IoMT integration to create networks of connected devices for continuous monitoring and real-time analysis (for example, smart insulin pumps); the ability of AR to increase surgical precision and support medical training; and cybersecurity measures to protect the sensitive health data transmitted by IoMT devices. Collaborative research with healthcare professionals, patients, and industry stakeholders will help ensure that the resulting solutions are user-friendly, technologically advanced, and aligned with real healthcare needs. Complementing this device-level focus, future bibliometric work should incorporate normalized cross-country benchmarking (per capita, per GDP, or per R&D expenditure) and co-authorship network analysis to further characterize collaboration dynamics.

### Ethics and consent

All ethical guidelines were strictly adhered to during the conduct of this research. Ethical approval and consent were not required, as the study analyses published bibliographic metadata and does not involve human participants or animals.

## Data Availability

Figshare: Comprehensive Dataset of Academic Publications on the Integration of Advanced Technologies in Medical Devices with PRISMA Documentation (2010-2024), https://doi.org/10.6084/m9.figshare.26412223.v2. ^46^ Data are available under the terms of the Creative Commons Attribution 4.0 International license (CC-BY 4.0).
