# Supplementary material for: Developing medical devices with emerging technologies: trends, challenges, and future directions
Source: F1000Res. 2026 Aug 17;13:1007. Originally published 2024 Sep 4. [Version 3] doi: 10.12688/f1000research.154869.3 (PMC13560011; doi:10.12688/f1000research.154869.3)
Supplement: Peer review history

# Reviewer report: Reviewer response for version 3

**DOI:** 10.5256/f1000research.207833.r512461

**Published:** 2026-08-21

**Authors:** Omid Panahi

**Affiliations:** 1 University of the People, Department of Healthcare Management, California, USA

This manuscript presents a bibliometric analysis of research on emerging technologies in medical devices, focusing primarily on artificial intelligence (AI), the Internet of Medical Things (IoMT), augmented/virtual reality, cybersecurity, big data, and embedded systems. The authors analyzed publications indexed in Scopus and Web of Science covering the period 2010–2024 and used Bibliometrix/Biblioshiny and VOSviewer to examine publication trends, geographical contributions, keyword evolution, and research clusters. The dataset includes 3,094 documents from 1,747 sources and provides a useful overview of the growth and thematic structure of research in this rapidly evolving field.

 The topic is timely and potentially valuable. The rationale and research questions are clearly presented, and the use of established bibliometric software provides a reasonable analytical framework. However, several methodological and interpretive issues remain important. In particular, the manuscript is presented as a “Systematic Review,” whereas its methodology is primarily bibliometric. Several elements expected in a conventional systematic review, such as formal study-quality/risk-of-bias assessment and critical appraisal of individual studies, are not applicable to the analysis as currently conducted. Therefore, the authors should clarify the study design and ensure that the terminology, methodology, and conclusions are fully aligned.

 1. Rationale and objectives

 Recommendation: Yes

 The rationale and objectives are sufficiently stated. The Introduction explains the increasing importance of AI, IoMT, AR, cybersecurity, and related technologies in medical devices and identifies several challenges, including privacy, infrastructure, and interoperability. The manuscript also explicitly formulates three research questions concerning regional contributions, technological trends, and implementation challenges.

 The research questions are therefore sufficiently clear and provide an understandable framework for the bibliometric analysis.

 Nevertheless, the authors should more explicitly distinguish between the objectives of a bibliometric analysis and those of a conventional systematic review. The current title and terminology may lead readers to expect a systematic synthesis of evidence concerning technological effectiveness or clinical outcomes, whereas the actual analysis primarily evaluates publication patterns, keywords, countries, authors, and research clusters.

 2. Methods and analysis – reproducibility

 Recommendation: Partly

 The Methods section provides useful information about the databases, general search concept, inclusion and exclusion criteria, publication period, duplicate removal, and software used. The revised manuscript has improved transparency by adding explicit inclusion/exclusion criteria and describing the use of Bibliometrix, Biblioshiny, and VOSviewer.

 However, the information is still not sufficient for complete independent replication.

 The authors should, at minimum: Provide the complete search strategy separately for Scopus and Web of Science, including the exact database fields searched, Boolean operators, truncations, filters, and date of each search. Report the exact dates on which the searches were conducted. Clearly describe how records were exported from each database and in which format. Explain the duplicate-removal procedure in sufficient technical detail. Clarify whether any manual screening was performed and, if so, how many reviewers were involved. Provide the complete keyword-cleaning/standardization procedure used before bibliometric analysis. Report the exact VOSviewer and Bibliometrix parameters used for the principal analyses, including thresholds, counting method, normalization method, clustering settings, and relevant software versions. Provide sufficient information to reproduce the country, keyword, and cluster analyses. These issues are particularly important because previous reviewers have also identified incomplete database-specific search strings, insufficient information concerning screening dates/processes, and inadequate description of duplicate removal as limitations to reproducibility.

 The authors have provided a PRISMA-style flow diagram, but PRISMA reporting alone does not make a bibliometric analysis equivalent to a conventional systematic review.

 3. Statistical analysis and interpretation

 Recommendation: Partly

 The selected bibliometric techniques are generally appropriate for the stated objective of mapping publication activity and research themes. The use of Bibliometrix/Biblioshiny and VOSviewer is reasonable for examining publication trends, keyword co-occurrence, and research clusters.

 However, the interpretation of several analyses should be strengthened.

 For example, publication counts are largely presented descriptively, and country-level output is reported mainly as absolute counts. Absolute publication counts can be strongly influenced by population size, economic resources, research infrastructure, and overall scientific productivity. Therefore, statements concerning the “emerging role” or relative importance of specific countries, particularly African countries, should be interpreted more cautiously unless appropriate normalization or contextual indicators are provided.

 Similarly, increasing keyword frequency demonstrates increasing use of terminology, but it does not necessarily establish technological advancement, clinical importance, or successful implementation. The authors should avoid interpreting bibliometric frequency as direct evidence of technological or clinical impact.

 The cluster analysis would also benefit from a more critical interpretation of cluster stability, evolution over time, and possible methodological artifacts.

 These concerns are consistent with previous reviewer observations regarding raw country counts, insufficient contextualization of keyword trends, and limited interpretation of cluster evolution.

 4. Conclusions

 Recommendation: Partly

 The conclusions are broadly consistent with the bibliometric findings, particularly regarding the increasing prominence of AI, IoT/IoMT, cybersecurity, and related technologies and the identification of major thematic clusters.

 However, some conclusions extend beyond what can be directly demonstrated by the bibliometric analysis. The analysis establishes publication trends and thematic patterns, but it cannot by itself demonstrate that these technologies improve patient outcomes, enhance clinical safety, accelerate personalized medicine, or produce successful real-world implementation.

 The authors should therefore distinguish clearly between:

 what the bibliometric data directly demonstrate;

 what can reasonably be inferred from the identified research trends; and

 broader implications or hypotheses requiring evidence from clinical and technological studies.

 The discussion should also be strengthened so that the title’s claims concerning “trends, challenges, and future directions” are supported by deeper critical analysis rather than primarily by publication counts and keyword patterns.

 A previous reviewer similarly identified a mismatch between the manuscript title and the predominantly bibliometric content and recommended either strengthening the technological discussion or modifying the title to reflect the bibliometric scope.

 5. Living Systematic Review

 Recommendation: Not applicable

 The manuscript does not appear to be a Living Systematic Review. No living-review methodology or predefined recurring search schedule is presented. Therefore, this criterion is not applicable.

 Major points that must be addressed

 The following issues should be considered essential for improving the scientific soundness of the manuscript: Clarify the study design and terminology. The authors should explicitly state whether this is a bibliometric analysis/bibliometric review rather than a conventional systematic review, and ensure that the title and manuscript terminology accurately reflect the methodology. Improve methodological reproducibility. Complete database-specific search strategies, search dates, data-export procedures, duplicate handling, and bibliometric parameters should be reported. Strengthen interpretation of bibliometric results. Absolute publication counts and keyword frequencies should not be interpreted as direct measures of technological, clinical, or scientific impact without appropriate qualification. Align conclusions with the evidence. Claims regarding patient outcomes, clinical effectiveness, implementation, and technological impact should be appropriately qualified because these outcomes were not directly assessed. Expand the critical discussion. The manuscript would benefit from a deeper evaluation of technological evolution, implementation barriers, cybersecurity, interoperability, ethical considerations, regulatory issues, and clinical adoption. Clarify the distinction between bibliometric mapping and systematic evidence synthesis. If the authors retain the term “systematic review,” they need to explain the methodological framework and limitations of this approach very carefully. Otherwise, reclassification as a “bibliometric analysis” or “bibliometric review” would be more appropriate.

 Overall assessment

 This is a timely and potentially useful study with a substantial bibliometric dataset and an appropriate general analytical framework. The rationale and research questions are clearly presented, and the results provide a useful map of research activity in emerging medical technologies.

 However, the manuscript currently has important methodological and interpretive limitations. The principal concern is the mismatch between the designation of the study as a systematic review and its predominantly bibliometric methodology. Additional improvements in reproducibility, interpretation of bibliometric indicators, and alignment between the conclusions and the actual evidence are required.

 I therefore recommend Major Revision. The manuscript could become a valuable contribution after the methodological terminology is clarified, reproducibility is strengthened, and the interpretation and conclusions are more closely restricted to what can be supported by the bibliometric evidence.

Are the rationale for, and objectives of, the Systematic Review clearly stated?

Yes

Is the statistical analysis and its interpretation appropriate?

Partly

If this is a Living Systematic Review, is the ‘living’ method appropriate and is the search schedule clearly defined and justified? (‘Living Systematic Review’ or a variation of this term should be included in the title.)

Not applicable

Are sufficient details of the methods and analysis provided to allow replication by others?

Partly

Are the conclusions drawn adequately supported by the results presented in the review?

Partly

Reviewer Expertise:

NA

I confirm that I have read this submission and believe that I have an appropriate level of expertise to state that I do not consider it to be of an acceptable scientific standard, for reasons outlined above.

# Reviewer report: Reviewer response for version 3

**DOI:** 10.5256/f1000research.207833.r511985

**Published:** 2026-08-20

**Authors:** Ibrahim Adabara

**Affiliations:** 1 Kampala International University - Western Campus, Bushenyi, Uganda

The authors have full addressed all concerns raised in the previous version based on the amended version, I confirmed that all concern had been addressed.

Are the rationale for, and objectives of, the Systematic Review clearly stated?

Yes

Is the statistical analysis and its interpretation appropriate?

Yes

If this is a Living Systematic Review, is the ‘living’ method appropriate and is the search schedule clearly defined and justified? (‘Living Systematic Review’ or a variation of this term should be included in the title.)

Not applicable

Are sufficient details of the methods and analysis provided to allow replication by others?

Partly

Are the conclusions drawn adequately supported by the results presented in the review?

Yes

Reviewer Expertise:

Artificial Intelligence in Healthcare, Cybersecurity Governance, Ethical and Agentic AI Systems, Digital Health Resilience, and Biomedical Informatics.My research explores the integration of agentic artificial intelligence frameworks for ethical governance and resilience in healthcare cybersecurity systems, focusing on the intersection of AI ethics, policy-driven digital health security, and autonomous decision intelligence in clinical and institutional environments.

I confirm that I have read this submission and believe that I have an appropriate level of expertise to confirm that it is of an acceptable scientific standard.

# Reviewer report: Reviewer response for version 3

**DOI:** 10.5256/f1000research.207833.r511984

**Published:** 2026-08-19

**Authors:** Soha Rawas

**Affiliations:** 1 Faculty of Science, Department of Mathematics and computer Science, Beirut Arab University, Beirut, Lebanon

No further revisions are required

Are the rationale for, and objectives of, the Systematic Review clearly stated?

Yes

Is the statistical analysis and its interpretation appropriate?

Partly

If this is a Living Systematic Review, is the ‘living’ method appropriate and is the search schedule clearly defined and justified? (‘Living Systematic Review’ or a variation of this term should be included in the title.)

Not applicable

Are sufficient details of the methods and analysis provided to allow replication by others?

Partly

Are the conclusions drawn adequately supported by the results presented in the review?

Partly

Reviewer Expertise:

Digital Health Technologies, Bibliometric Analysis,  Healthcare Data Analytics, IOMT, Cloud computing

I confirm that I have read this submission and believe that I have an appropriate level of expertise to confirm that it is of an acceptable scientific standard.

# Reviewer report: Reviewer response for version 3

**DOI:** 10.5256/f1000research.207833.r511986

**Published:** 2026-08-19

**Authors:** Shehu Mohammed

**Affiliations:** 1 School of Computer Applications, Lovely Professional University, Phagwara, Punjab, India; 2 Department of Computer Science, Abdu Gusau Polytechnic, Talata Mafara, Zamfara, Nigeria

The authors have significantly addressed my previous comments, particularly regarding the clarification of the bibliometric study design, methodological limitations, analytical depth, removal of duplicated content, and language quality. I appreciate the revisions made.

 One methodological point would benefit from further clarification: although the revised manuscript states that full Scopus and Web of Science search strings have been provided, the Methods section does not clearly distinguish the database-specific search syntax and field restrictions. In addition, the reported May 2024 search date should be clarified in relation to the stated 2010–2024 publication period. Clarifying these points would improve the reproducibility of the study.

Are the rationale for, and objectives of, the Systematic Review clearly stated?

Yes

Is the statistical analysis and its interpretation appropriate?

Yes

If this is a Living Systematic Review, is the ‘living’ method appropriate and is the search schedule clearly defined and justified? (‘Living Systematic Review’ or a variation of this term should be included in the title.)

Not applicable

Are sufficient details of the methods and analysis provided to allow replication by others?

Partly

Are the conclusions drawn adequately supported by the results presented in the review?

Yes

Reviewer Expertise:

Artificial Intelligence in Healthcare, Internet of Medical Things (IoMT), Biomedical Engineering, Digital Health Technologies, Medical Device Innovation, Machine Learning Applications in Healthcare, Healthcare Data Analytics, and Cybersecurity in Medical Systems.

I confirm that I have read this submission and believe that I have an appropriate level of expertise to confirm that it is of an acceptable scientific standard, however I have significant reservations, as outlined above.

# Reviewer report: Reviewer response for version 2

**DOI:** 10.5256/f1000research.193492.r486955

**Published:** 2026-07-01

**Authors:** N. Chaithra Chaithra

**Affiliations:** 1 JAIN (Deemed to be University), Bangalore, Karnataka, India

This manuscript presents a bibliometric review of emerging technologies in medical devices using publications retrieved from the Scopus and Web of Science databases.

 The study provides a useful overview of publication trends, keyword evolution, country-wise scientific production, and research clusters related to AI, IoMT, AR, cybersecurity, and embedded systems. The methodology is generally well described, and the bibliometric analysis is systematically presented.

 However, I believe the conclusions are only **partly supported** by the results presented because there is a noticeable mismatch between the manuscript's title and its actual content.

 The title suggests that the review will comprehensively discuss **trends, challenges, and future directions** in the development of medical devices. In practice, a substantial portion of the paper, particularly Sections 4 to 7, is devoted to bibliometric analyses such as keyword frequencies, publication counts, citation patterns, leading authors, and country contributions. While these analyses provide valuable information regarding research activity, they do not sufficiently explain the technological evolution of medical devices or critically evaluate the emerging trends highlighted in the title.

**Major revisions required to improve scientific soundness:**

1. Expand the discussion beyond the bibliometric findings to provide a deeper critical analysis of the technological evolution of medical devices.
2. Provide more detailed coverage of emerging technologies such as AI, Machine Learning, Big Data, IoMT, AR/VR, and cybersecurity, with greater emphasis on their real-world applications, innovations, and implementation challenges.
3. Include representative case studies or detailed examples of advanced medical devices to better support the discussion of current technological trends and their practical impact.
4. Strengthen the discussion on challenges and future directions by addressing technical, regulatory, interoperability, ethical, and clinical adoption issues in greater depth.
5. Reduce the repeated references to the Scopus and Web of Science databases throughout the manuscript and instead focus on interpreting the findings and their implications for medical device research.
6. Ensure consistency between the manuscript title and its content. If the primary contribution is a bibliometric analysis, the title should better reflect that scope. Otherwise, the discussion should be expanded to comprehensively address trends, challenges, and future directions.
7. Clearly identify and discuss the research gap addressed by this review. The novelty and contribution of the manuscript should be explicitly stated in comparison with existing review articles.
8. Expand the cybersecurity discussion to better reflect current challenges in AI-enabled medical devices. This should include security implications of Generative AI, adversarial attacks, distributed denial-of-service (DDoS) attacks, man-in-the-middle (MITM) attacks, and other emerging threats. The authors should also discuss modern privacy-preserving and secure AI approaches such as differential privacy, federated learning, and edge computing, which are becoming increasingly relevant for intelligent healthcare systems.
9. Include a dedicated section discussing ethical, legal, and regulatory considerations to improve the credibility and completeness of the review. This section should cover evolving regulations related to AI governance, cybersecurity, medical device compliance, and patient data privacy.

Overall, this is a well-structured paper with a solid bibliometric foundation. With a deeper critical discussion and broader coverage of emerging technologies, cybersecurity, ethics, and regulatory aspects, the manuscript could become a significantly stronger and more credible review that provides greater value to researchers and practitioners in the field.

Are the rationale for, and objectives of, the Systematic Review clearly stated?

Yes

Is the statistical analysis and its interpretation appropriate?

Yes

If this is a Living Systematic Review, is the ‘living’ method appropriate and is the search schedule clearly defined and justified? (‘Living Systematic Review’ or a variation of this term should be included in the title.)

Yes

Are sufficient details of the methods and analysis provided to allow replication by others?

Yes

Are the conclusions drawn adequately supported by the results presented in the review?

Partly

Reviewer Expertise:

Artificial Intelligence (AI), Machine Learning (ML), Deep Learning (DL), Generative AI, AI in Healthcare, Medical AI, Intelligent Healthcare Systems

I confirm that I have read this submission and believe that I have an appropriate level of expertise to confirm that it is of an acceptable scientific standard, however I have significant reservations, as outlined above.

# Response

**Published:** 2026-07-31

**Authors:** Achraf akkaoui

**Affiliations:** Universite Hassan 1er Faculte des Sciences et Techniques Settat, Settat, Chaouia-Ouardigha, Morocco

We thank the reviewer for the thorough and encouraging review.

1. *Technological evolution.* §8 now analyses the shift toward connected, AI-enabled, software-defined devices, beyond the bibliometric metrics.
2. *Technology coverage.* §3 and §8 now include concrete applications and challenges for each technology.
3. *Case studies.* §8.2 adds real-device examples (IDx-DR, AI-enabled ECG, closed-loop insulin delivery, robotic and AR-assisted surgery).
4. *Challenges and future directions.* Technical, regulatory, interoperability, ethical, and adoption issues are now treated explicitly across §8, §9, and Future Directions.
5. *Database repetition.* Redundant Scopus/Web of Science mentions have been trimmed in favour of interpretation.
6. *Title–content consistency.* We retained the title and, as the reviewer proposed, expanded the discussion so that trends, challenges, and future directions are addressed comprehensively; the bibliometric methodology is clarified in the abstract and Methods.
7. *Research gap and novelty.* The Introduction now states the gap explicitly: unlike prior single-technology studies, this work maps the convergence of AI, IoMT, AR/VR, embedded systems, and cybersecurity, and foregrounds African contributions.
8. *Cybersecurity.* §8.3 now covers generative-AI risks, adversarial attacks, DDoS and MITM threats, and privacy-preserving approaches (differential privacy, federated learning, edge computing).
9. *Ethics and regulation.* A new §9 addresses AI governance (EU AI Act, FDA AI/ML SaMD), device regulation and standards (MDR, IEC 62304, IEC 81001-5-1), and data privacy (GDPR, HIPAA, Morocco CNDP/Law 09-08).

# Reviewer report: Reviewer response for version 2

**DOI:** 10.5256/f1000research.193492.r494840

**Published:** 2026-07-01

**Authors:** Soha Rawas

**Affiliations:** 1 Faculty of Science, Department of Mathematics and computer Science, Beirut Arab University, Beirut, Lebanon

This manuscript presents a bibliometric analysis of emerging technologies (AI, IoMT, AR, cybersecurity) in medical device development, analyzing 3,094 publications from 2010-2024. While the study addresses a timely topic and demonstrates substantial data collection effort, several critical issues require attention before indexing.

**Major Concerns**

 The study is described throughout as a "Systematic Review," yet the methodology is clearly that of a bibliometric analysis. Key systematic review components, protocol registration, quality/risk-of-bias assessment, critical appraisal of included studies, and data extraction beyond metadata, are absent. The authors should reclassify this as a "Bibliometric Analysis" or "Bibliometric Review" throughout the manuscript.

 The methodology lacks completeness for replication. The authors must provide full database-specific search strings with field restrictions, specify screening dates, describe the screening process (number of reviewers, independence, disagreement resolution), and detail duplicate removal criteria.

 Country production data are presented as raw counts without normalization (population, GDP, or research investment). Keyword trends show increased usage but lack contextual explanation. The cluster analysis describes three research areas but does not critically examine their evolution, research gaps, or potential artifact.

 The Discussion would benefit from comparative evaluation of technologies and practical implementation challenges.

 The Future Directions section contains identical text repeated on pages 12-13 and requires revision.

 The analysis lacks depth, no collaborative patterns, trend data showing growth trajectory, or comparative metrics to substantiate the "emerging role" claim.

 This manuscript requires substantial revision, particularly reclassification of study type and improved methodological transparency

Are the rationale for, and objectives of, the Systematic Review clearly stated?

Yes

Is the statistical analysis and its interpretation appropriate?

Partly

If this is a Living Systematic Review, is the ‘living’ method appropriate and is the search schedule clearly defined and justified? (‘Living Systematic Review’ or a variation of this term should be included in the title.)

Not applicable

Are sufficient details of the methods and analysis provided to allow replication by others?

Partly

Are the conclusions drawn adequately supported by the results presented in the review?

Partly

Reviewer Expertise:

Digital Health Technologies, Bibliometric Analysis,  Healthcare Data Analytics, IOMT, Cloud computing

I confirm that I have read this submission and believe that I have an appropriate level of expertise to state that I do not consider it to be of an acceptable scientific standard, for reasons outlined above.

# Response

**Published:** 2026-07-31

**Authors:** Achraf akkaoui

**Affiliations:** Universite Hassan 1er Faculte des Sciences et Techniques Settat, Settat, Chaouia-Ouardigha, Morocco

We thank the reviewer for the detailed evaluation, which has strengthened the manuscript.

1. *Reclassification.* The study is now framed as a bibliometric analysis in the abstract and Methods; we state that protocol registration, critical appraisal, and risk-of-bias assessment are not part of this design. The title is retained and the final label deferred to the editors.
2. *Reproducibility.* Full database-specific search strings, search date, independent screening with disagreement resolution, and duplicate-removal criteria are now provided.
3. *Country normalization.* §4.2 now interprets rankings against population and R&D capacity, adds a normalized perspective, and flags raw counts as a limitation.
4. *Keyword context.* §4.1 now explains the drivers of the observed trends (deep-learning breakthroughs, telehealth expansion, rising cyber-threats).
5. *Cluster analysis.* §4.3 now discusses cluster evolution, under-represented topics, and the artefact introduced by the co-occurrence threshold.
6. *Comparative evaluation.* Added in §8 (Table 5).
7. *Future Directions.* Duplicated text removed.
8. *Depth.* A new §4.4 interprets collaboration metrics and the 27.8% growth trajectory to substantiate the "emerging role" claim.

# Reviewer report: Reviewer response for version 2

**DOI:** 10.5256/f1000research.193492.r479319

**Published:** 2026-05-07

**Authors:** Shehu Mohammed

**Affiliations:** 1 School of Computer Applications, Lovely Professional University, Phagwara, Punjab, India; 2 Department of Computer Science, Abdu Gusau Polytechnic, Talata Mafara, Zamfara, Nigeria

This manuscript presents a timely and relevant study on the integration of emerging technologies, including artificial intelligence (AI), Internet of Medical Things (IoMT), augmented reality (AR), and cybersecurity, in medical device development. The rationale and objectives are clearly stated, and the study is supported by a substantial dataset and appropriate bibliometric tools, providing useful insights into global research trends and technological advancements.

 However, several aspects require improvement to enhance the scientific rigor and clarity of the manuscript. First, the study is described as a systematic review, whereas the methodology aligns more closely with a bibliometric analysis. This distinction should be clarified throughout the manuscript to ensure methodological accuracy.

 Second, the methodological description lacks sufficient detail to allow full reproducibility. Specifically, the authors should provide complete database-specific search queries, clearly indicate the search dates, and describe the screening and selection process in greater detail.

 Third, the manuscript does not include any form of study quality or risk-of-bias assessment, which limits its rigor if positioned as a systematic review. This should either be incorporated or explicitly acknowledged as a limitation.

 Additionally, the discussion section is largely descriptive and would benefit from deeper critical analysis. The authors are encouraged to include comparative evaluations of the technologies discussed, highlight practical implementation challenges, and provide more analytical insights.

 There is also some redundancy in the “Future Directions” section, which should be revised to remove duplicated content and improve conciseness. Minor grammatical inconsistencies and informal phrasing are present and should be corrected to ensure a consistent academic tone.

 Overall, the manuscript makes a valuable contribution to the field and, with these revisions, has strong potential for indexing.

Are the rationale for, and objectives of, the Systematic Review clearly stated?

Yes

Is the statistical analysis and its interpretation appropriate?

Yes

If this is a Living Systematic Review, is the ‘living’ method appropriate and is the search schedule clearly defined and justified? (‘Living Systematic Review’ or a variation of this term should be included in the title.)

Not applicable

Are sufficient details of the methods and analysis provided to allow replication by others?

Partly

Are the conclusions drawn adequately supported by the results presented in the review?

Yes

Reviewer Expertise:

Artificial Intelligence in Healthcare, Internet of Medical Things (IoMT), Biomedical Engineering, Digital Health Technologies, Medical Device Innovation, Machine Learning Applications in Healthcare, Healthcare Data Analytics, and Cybersecurity in Medical Systems.

I confirm that I have read this submission and believe that I have an appropriate level of expertise to confirm that it is of an acceptable scientific standard, however I have significant reservations, as outlined above.

# Response

**Published:** 2026-07-31

**Authors:** Achraf akkaoui

**Affiliations:** Universite Hassan 1er Faculte des Sciences et Techniques Settat, Settat, Chaouia-Ouardigha, Morocco

We thank the reviewer for the constructive assessment.

1. *Study type.* We agree the methodology is a bibliometric analysis and have reframed it as such in the abstract and Methods. The title is retained; "Systematic Review" was the closest category offered at submission, and we defer the final article-type label to the editors.
2. *Reproducibility.* The Methods now give the full Scopus and Web of Science search strings, the search date, the screening and de-duplication procedure, and the PRISMA flow (Figure 1).
3. *Risk of bias.* We now state explicitly that, as a bibliometric analysis of metadata, no critical appraisal or risk-of-bias assessment was performed, and we acknowledge this as a design limitation.
4. *Discussion depth.* We added a comparative evaluation of the technologies (Table 5) and expanded analysis of implementation challenges, with case studies.
5. *Future Directions.* The duplicated text has been removed.
6. *Language.* Informal phrasing and grammar have been corrected throughout.

# Reviewer report: Reviewer response for version 1

**DOI:** 10.5256/f1000research.169950.r435467

**Published:** 2025-12-11

**Authors:** Ibrahim Adabara

**Affiliations:** 1 Kampala International University - Western Campus, Bushenyi, Uganda

This manuscript presents a **comprehensive and well-structured systematic review** that examines the integration of emerging technologies in medical device development, focusing on **Artificial Intelligence (AI)**, **the Internet of Medical Things (IoMT)**, **Augmented Reality (AR)**, and **cybersecurity**.

 The study’s **rationale is clearly justified**, and the **research questions are well defined**.

 The **methodological approach**, which combines Scopus and Web of Science datasets with bibliometric tools such as **Bibliometrix**, **Biblioshiny**, and **VOSviewer**, is robust and enhances the credibility of the analysis.

 The **visualizations are informative** and effectively illustrate key trends, collaborations, and thematic clusters within this growing field.

 The **discussion provides valuable insights** into global research dynamics, particularly through its emphasis on **Africa’s and Morocco’s emerging roles in healthcare innovation**. This regional focus adds originality and significance to the work.

 However, the **methodology section would benefit from more detailed explanations** regarding inclusion and exclusion criteria, data quality assessment, and management of potential publication bias.

 The **discussion could also include a deeper critical reflection** on the limitations and ethical implications of applying AI and cybersecurity within clinical settings.

 Overall, this article makes a **significant and timely contribution** to understanding how advanced technologies are transforming the medical device industry. With **minor revisions aimed at improving methodological transparency and analytical depth**, it has strong potential to serve as an important reference for researchers and policymakers working at the intersection of innovation, digital health, and biomedical engineering.

Are the rationale for, and objectives of, the Systematic Review clearly stated?

Yes

Is the statistical analysis and its interpretation appropriate?

Yes

If this is a Living Systematic Review, is the ‘living’ method appropriate and is the search schedule clearly defined and justified? (‘Living Systematic Review’ or a variation of this term should be included in the title.)

Not applicable

Are sufficient details of the methods and analysis provided to allow replication by others?

Partly

Are the conclusions drawn adequately supported by the results presented in the review?

Yes

Reviewer Expertise:

Artificial Intelligence in Healthcare, Cybersecurity Governance, Ethical and Agentic AI Systems, Digital Health Resilience, and Biomedical Informatics.My research explores the integration of agentic artificial intelligence frameworks for ethical governance and resilience in healthcare cybersecurity systems, focusing on the intersection of AI ethics, policy-driven digital health security, and autonomous decision intelligence in clinical and institutional environments.

I confirm that I have read this submission and believe that I have an appropriate level of expertise to confirm that it is of an acceptable scientific standard, however I have significant reservations, as outlined above.

## References

[1] : Developing medical devices with emerging technologies: trends, challenges, and future directions. *F1000Research* .2024;13: 10.12688/f1000research.154869.1 10.12688/f1000research.154869.1 PMC1356001142728902

# Response

**Published:** 2026-02-11

**Authors:** Achraf akkaoui

**Affiliations:** Universite Hassan 1er Faculte des Sciences et Techniques Settat, Settat, Chaouia-Ouardigha, Morocco

We sincerely thank the reviewer for the careful evaluation of our manuscript and for the constructive recommendations. We appreciate the positive assessment of the study rationale, research questions, methodological framework, and the clarity of the bibliometric visualisations, as well as the recognition of the regional contribution highlighting Africa and Morocco.

 In response to the reviewer’s reservations, we have revised the manuscript to improve methodological transparency and analytical depth. Specifically, the Methods section now includes explicit inclusion and exclusion criteria, clearer justification of data processing requirements, and an added statement on potential publication bias, including mitigation through combining Scopus and Web of Science while acknowledging remaining limitations. The Discussion has also been strengthened with a deeper critical reflection on study limitations and on ethical and clinical implications of AI, IoMT, AR, and cybersecurity, including issues of transparency, accountability, privacy, bias, and the need for user training and robust governance.
